# Discovery and Heterologous Production of New Cyclic Depsibosamycins

**DOI:** 10.3390/microorganisms9071396

**Published:** 2021-06-28

**Authors:** Marc Stierhof, Maksym Myronovskyi, Josef Zapp, Andriy Luzhetskyy

**Affiliations:** 1 Department of Pharmaceutical Biotechnology, Saarland University, 66123 Saarbruecken, Germany; marc.stierhof@uni-saarland.de (M.S.); maksym.myronovskyi@uni-saarland.de (M.M.); 2 Department of Pharmaceutical Biology, Saarland University, 66123 Saarbruecken, Germany; j.zapp@mx.uni-saarland.de; 3 AMEG Department, Helmholtz Institute for Pharmaceutical Research Saarland, 66123 Saarbrücken, Germany

**Keywords:** bosamycin, *Streptomyces*, heterologous expression, NRPS, cyclic peptide

## Abstract

*Streptomyces* are producers of valuable secondary metabolites with unique scaffolds that perform a plethora of biological functions. Nonribosomal peptides are of special interest due to their variety and complexity. They are synthesized by nonribosomal peptide synthetases, large biosynthetic machineries that are encoded in the genome of many *Streptomyces* species. The identification of new peptides and the corresponding biosynthetic gene clusters is of major interest since knowledge can be used to facilitate combinatorial biosynthesis and chemical semisynthesis of natural products. The recently discovered bosamycins are linear octapeptides with an interesting 5-OMe tyrosine moiety and various modifications at the *N*-terminus. In this study, the new cyclic depsibosamycins B, C, and D from *Streptomyces aurantiacus* LU19075 were discovered. In comparison to the linear bosamycins B, C, and D, which were also produced by the strain, the cyclic depsibosamycins showed a side-chain-to-tail lactonization of serine and glycine, leading to a ring of four amino acids. In silico identification and heterologous expression of the depsibosamycin (*dbm*) gene cluster indicated that the cyclic peptides, rather than the linear derivatives, are the main products of the cluster.

## 1. Introduction

*Streptomyces* are known to produce valuable secondary metabolites with a plethora of unique structural classes [1,2]. These compounds often express a variety of biological functions, which are the product of centuries of evolutionary design to develop a selective advantage for the strain [3]. The enormous structural complexity of natural products from *Streptomyces,* which are generated by enzymatic biosynthetic machinery, can be recreated only with difficulty by chemical synthesis. Therefore, a detailed understanding of the biosynthetic origin of natural products is of major interest to facilitate combinatorial biosynthesis and semisynthetic approaches, which are important tools in drug development.

Nonribosomal peptide synthetases (NRPSs) generate highly diverse peptides and are a coveted target for biosynthetic engineering [4,5]. Nonribosomal peptides (NRPs) are synthesized by modular enzymes. Individual modules consist of a minimal set of three domains: the adenylation domain (A), thiolation domain (T), and condensation domain (C). Biosynthesis is initiated by the A-domain, which specifically activates natural or modified amino acids. The activated amino acid is tethered to the pantetheinyl arm of the T-domain. The condensation reaction with another amino acid, selected by an adjacent module, is specifically catalyzed by the C-domain. Each module can contain additional domains to generate D-amino acids, *N*-methylated peptide bonds, heterocyclic amino acids, etc. [6]. The mature peptide is cleaved from the NRPS assembly line by the thioesterase domain (TE), resulting in a linear or cyclic peptide [6,7].

The recent development of gene sequencing, bioinformatics, and genomic tools has enabled the targeted identification, expression, or activation of unique biosynthetic gene clusters (BGCs), which is a major leap forward in the discovery of new natural products [8]. By targeting novel NRPS architectures, Xu et al. recently discovered the bosamycin gene cluster (*bsm* cluster), encoding the production of new linear octapeptides named bosamycins. The NRPS assembly line contains eight modules leading to the following peptide sequence: L-Tyr, D-Tyr, L-Leu, L-*erythro*-*β*-OH-Asp, L-Ser, 5-MeO-D-Tyr, L-Leu, and Gly. The 5-OH tyrosine moiety is generated by a stand-alone NRPS with a unique P450 domain, but no C-domain is present in the module. The octapeptide chain is further modified by the two separate genes encoding an O-methyltransferase and a dioxygenase, leading to 5-OMe tyrosine and *erythro*-*β*-OH asparagine. Bosamycin E showed slight inhibitory activity towards SHP2 (Src homology 2-containing protein tyrosine phosphatase 2), while other derivatives had no biological activity [9].

**Figure 1:**
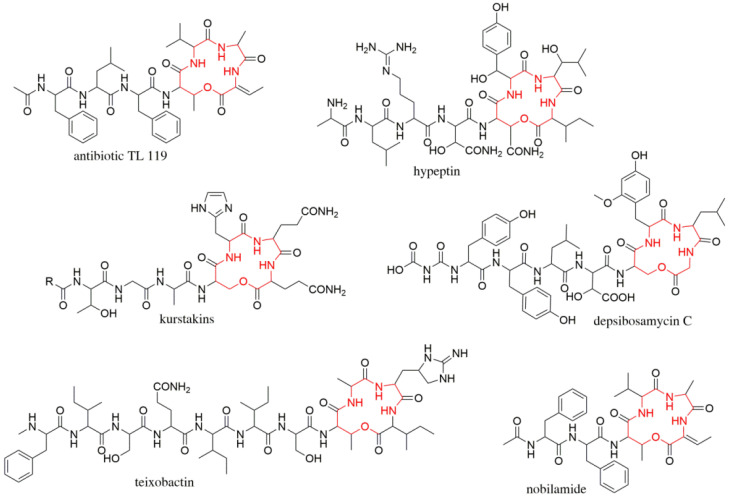
Structure comparison of compounds with a similar side-chain-to-tail lactone ring (red) formed by an intramolecular reaction of *β*-hydroxy amino acids and the *C*-terminus.

Herein, we describe the discovery of the new cyclic depsibosamycins B, C, and D. The compounds were identified by metabolic profiling of *Streptomyces aurantiacus* LU19075 and characterized by MS/MS fragmentation and NMR spectroscopy. Structure elucidation studies revealed a side-chain-to-tail lactonization of serine and glycine within the structure depsibosamycin. This led to a ring including four amino acids, a feature that has also been described for hypeptin, teixobactin, and others (Figure 1). In this paper, we postulate that the cyclic depsibosamycins, rather than the linear ones, are the main products of the BGC. This was supported by the results of the heterologous expression experiments and comparisons of the production rate.

## 2. Materials and Methods

### 2.1. General Procedures

All strains and bacterial artificial chromosomes (BACs) used in this work are listed in Table S1. *Escherichia coli* strains were cultured in LB medium [10]. *Streptomyces* strains were grown on soya flour mannitol agar (MS agar) [11] or in liquid tryptic soy broth (TSB; Sigma-Aldrich, St. Louis, MO, USA) for cultivation. Liquid DNPM medium (40 g/L dextrin, 7.5 g/L soytone, 5 g/L baking yeast, and 21 g/L MOPS, pH 6.8) was used for secondary metabolite expression. The antibiotics kanamycin, apramycin, and nalidixic acid were supplemented when required.

### 2.2. Metabolite Extraction and Analysis by Mass Spectrometry (MS)

*S. aurantiacus* LU19075 was grown in 20 mL of TSB in a 100 mL baffled flask for one day, and 1 mL of the culture was inoculated into 100 mL of DNPM production medium in a 500 mL baffled flask. Cultures were grown for 6 days at 28 °C and 180 rpm in an Infors multitron shaker (Infors AG, Basel, Switzerland). Metabolites were extracted from the supernatant with butanol, concentrated to dryness, and dissolved in 1 mL methanol (MeOH). One microliter was separated on a Dionex Ultimate 3000 UPLC system (Thermo Fisher Scientific, Waltham, MA, USA) equipped with an ACQUITY UPLC BEH C18 1.7 µm column (30, 50, or 100 mm, Waters Corporation, Milford, MA, USA) using a linear gradient of 5–95 vol% aqueous acetonitrile (ACN) with 0.1% formic acid (FA) at a flow rate of 0.6 mL/min and a column oven temperature of 45 °C. Sample analysis was carried out on a coupled PDA detector followed by an amaZon speed (Bruker, Billerica, MA, USA) for production control. High-resolution masses were obtained from either an LTQ Orbitrap XL mass spectrometer (Thermo Fisher Scientific, Waltham, MA, USA) or a MaXis high-resolution LC-QTOF system (Bruker, Billerica, MA, USA) using positive ionization mode and mass range detection of *m*/*z* 200 to 2000. Data analysis was performed using Compass Data Analysis v. 4.1 (Bruker) and Xcalibur v. 3.0 (Thermo Fisher Scientific).

### 2.3. Nuclear Magnetic Resonance (NMR) Spectroscopy and Optical Rotation (OD)

The NMR spectra of *β*-OH-Asp and bosamycin D were recorded on a Bruker Avance, UltraShield 500 MHz (Bruker, BioSpin GmbH, Rheinstetten, Germany) equipped with a 5 mm BBO probe at 298 K. All other NMR spectra were acquired on a Bruker Avance III, Ascent 700 MHz spectrometer at 298 K equipped with a 5 mm TCI cryoprobe. The chemical shifts (δ) were reported in parts per million (ppm) relative to tetramethylsilane (TMS). As solvents, deuterated CD_3_OD (δ_H_ 3.31 ppm, δ_C_ 49.15 ppm), DMSO-*d*6 (δ_H_ 2.50 ppm, δ_C_ 39.51 ppm), and D_2_O (δ_H_ 4.75 ppm) from Deutero (Kastellaun, Germany) were used. Edited-HSQC, HSQC-TOCSY, HMBC, ^1^H-^1^H COSY, ROESY, NOESY, and N-HSQC spectra were recorded using standard pulse programs from TOPSPIN v.3.6 software. Optical rotations were measured using a Perkin Elmer Polarimeter Model 241 (Überlingen, Deutschland).

### 2.4. Isolation of Bosamycin B–D and Depsibosamycin C

*S. aurantiacus* LU19075 was grown in 10 L of DNPM production medium and extracted with butanol. The dry crude extract was dissolved in 100 mL of methanol. The isolation process was guided by LC-MS to identify fractions. The extract was purified on an Isolera^TM^ One flash purification system equipped with a SNAP Ultra C_18_ 400 g (Biotage, Uppsala, Sweden) using a gradient of 5–30 vol% aqueous methanol for 3 column volumes (CV) followed by 30–95 vol% aqueous methanol for 1.5 CV at a flow rate of 100 mL/min and UV detection at 210 and 280 nm. Further impurities were removed by size exclusion chromatography (SEC; stationary phase: Sephadex-LH20) with isocratic elution using 100% methanol.

The crude material was dissolved in methanol, and reversed-phase HPLC was carried out on a Waters Autopurification System (Waters Corporation, Milford, MA, USA) equipped with a SQD2-MS-Detector and equipped with a preparative VP 250/21 NUCLEODUR C_18_ HTec 5 µm column (MACHERY NAGEL, Düren, Germany) using a gradient of 55–75 vol% aqueous methanol with 0.1% FA for 12 min at a flow rate of 20 mL/min.

The final purification step was carried out on an Agilent Infinity 1100 series reversed-phase HPLC system equipped with a Synergi^TM^ 4 µm Fusion-RP C_18_ 80 Å 250 × 10 mm column (Phenomenex, Torrance, CA, USA) using a gradient of 31–33 vol% aqueous ACN with 0.1% FA, a flow rate of 4 mL/min, and an oven temperature of 20 °C. The compounds were detected with a PDA detector at 210 and 280 nm at RT = 10.06 min (bosamycin B), 10.29 min (bosamycin C), 10.69 min (bosamycin D), and 11.74 min (depsibosamycin C).

#### 2.4.1. Bosamycin B

White powder; 1.6 mg; ${\left[\alpha \right]}_{D}^{20}$-38 (c 0.06, MeOH); UV (37% ACN in H_2_O + 0.1% FA) λ_max_ (log ε) 200 (4.25), 276 (2.93) nm; ^1^H and ^13^C NMR data, see Table S3; ESI-TOF-MS *m*/*z* 1082.4650 [M + H]^+^ (calc. for C_50_H_68_N_9_O_18_ 1082.4677), see Figure S1.

#### 2.4.2. Bosamycin C

White powder; 6.8 mg; ${\left[\alpha \right]}_{D}^{20}$-3 (c 0.27, DMSO); UV (37% ACN in H_2_O + 0.1% FA) λ_max_ (log ε) 200 (3.82), 276 (2.62) nm; ^1^H and ^13^C NMR data, see Table S4; ESI-TOF-MS *m*/*z* 1126.4595 [M + H]^+^ (calc. for C_51_H_68_N_9_O_20_ 1126.4575), see Figure S1.

#### 2.4.3. Bosamycin D

White powder; 9.1 mg; ${\left[\alpha \right]}_{D}^{20}$-31 (c 0.21, MeOH); UV (37% ACN in H_2_O + 0.1% FA) λ_max_ (log ε) 200 (3.96), 276 (2.95) nm; ^1^H and ^13^C NMR data, see Table S5; ESI-TOF-MS *m*/*z* 1081.4718 [M + H]^+^ (calc. for C_51_H_69_N_8_O_18_ 1081.4724), see Figure S1.

#### 2.4.4. Depsibosamycin C

White powder; 4.8 mg; ${\left[\alpha \right]}_{D}^{20}$-13 (c 0.24, DMSO); UV (42% ACN in H_2_O + 0.1% FA) λ_max_ (log ε) 200 (3.95), 276 (2.65) nm; ^1^H and ^13^C NMR data, see Table S2; ESI-TOF-MS *m*/*z* 1108.4496 [M + H]^+^ (calc. for C_51_H_66_N_9_O_19_ 1108.4470), see Figure S1.

### 2.5. Marfey’s Analysis

Bosamycin C was hydrolyzed in 100 µL 6 N HCl at 110 °C for 1 h. While cooling, the sample was dried for 15 min under nitrogen and dissolved in 110 mL water, and 50 µL of each sample was transferred into 1.5 mL Eppendorf tubes. To the hydrolysate, 20 µL of 1 N NaHCO_3_ and 20 µL of 1% L-FDLA (*N*^α^-(5-Fluoro-2,4-dinitrophenyl)-L-leucinamide) or D-FDLA in acetone were added. The amino acid standards were prepared the same way using only L-FDLA. The reaction mixtures were incubated at 40 °C for 90 min at 700 rpm and subsequently quenched with 2 N HCl to stop the reaction. The samples were diluted with 300 µL ACN, and 1 µL aliquots were analyzed by a MaXis high-resolution LC-QTOF system using aqueous ACN with 0.1 vol% FA and an adjusted gradient of 5–10 vol% in 2 min, 10–25 vol% in 13 min, 25–50 vol% in 7 min, and 50–95 vol% in 2 min. Sample detection was carried out at 340 nm. β-Hydroxyaspartic acid diastereomers were synthesized from *cis-* and *trans*-epoxysuccinic acid according to the literature [12].

#### 2.5.1. DL-Erythro-Hydroxyaspartic Acid

White crystals; yield 24%; ^1^H NMR (500 MHz, D_2_O, pH = 4) δ: 4.47 (d, *J* = 3.2, 1H), 4.17 (d, *J* = 3.2, 1H) (Figure S37); ESI-TOF-MS *m*/*z* 150.0398 [M + H]^+^ (calc. for C_4_H_8_NO_5_ 150.0403).

#### 2.5.2. DL-Threo-Hydroxyaspartic Acid

White crystals, yield 42%, ^1^H NMR (500 MHz, D_2_O, pH = 12) δ: 4.35 (d, *J* = 2.2, 1H), 3.60 (d, *J* = 2.2, 1H) (Figure S38); ESI-TOF-MS *m*/*z* 150.0398 [M + H]^+^ (calc. for C_4_H_8_NO_5_ 150.0403).

### 2.6. Isolation and Manipulation of DNA

BAC extraction from a *S. aurantiacus* LU19075 constructed genomic library (Intact Genomics, St. Louis, MO, USA), DNA manipulation, *E. coli* transformation, and *E. coli/Streptomyces* intergeneric conjugation were performed according to standard protocols [10,11,13,14]. Plasmid DNA was purified with the BACMAX™ DNA Purification Kit (Lucigen, Middleton, WI, USA). Restriction endonucleases were used according to the manufacturer’s recommendations (New England Biolabs, Ipswich, MA, USA).

### 2.7. Genome Mining and Bioinformatics Analysis

An *S. aurantiacus* LU19075 genome BAC library using the pSMART vector with apramycin resistance gene was constructed and sequenced by Intact Genomics (Missouri, St. Louis, USA). The genome was screened for secondary metabolite biosynthetic gene clusters using the antiSMASH online tool with loose settings (Available online: https://antismash.secondarymetabolites.org/#!/start) [15] (accessed on 3 June 2021). Analysis of genetic data was performed using Geneious 11.0.3 [16]. The DNA sequence of the *dbm* gene cluster from LU19075 was deposited into GenBank under the accession number MW740414.

## 3. Results and Discussion

### 3.1. Identification and Structural Elucidation of Depsibosamycins B, C, and D

**Figure 2:**
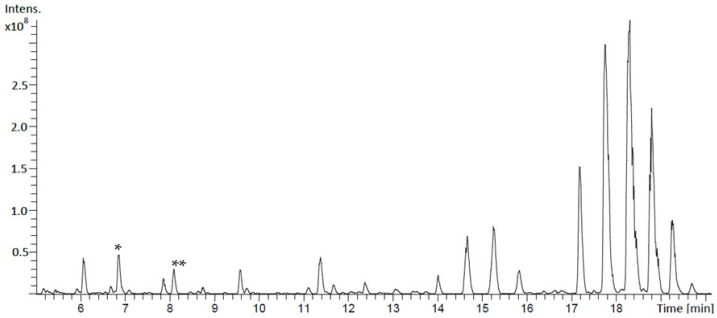
LC-MS chromatogram of the butanol extract of *Streptomyces aurantiacus* LU19075 cultivated in DNPM medium showing the peaks of bosamycins B, C, and D (*) and depsibosamycins B, C, and D (**). In addition to that, nactins (17–20 min) and nactin degradation products (9–16 min) were identified.

We screened several strains from our strain library for the production of new compounds by comparing UV/VIS spectra, retention times, MS/MS fragmentation patterns, and HRMS data with those from natural product databases, such as “Dictionary of Natural Products” (DNP) [17]. By this, *Streptomyces aurantiacus* LU19075 caught our attention due to the production of a large quantity of compounds that were identified as well described members of the nactin family (Figure 2) [18]. Furthermore, six unknown compounds eluted in two groups at RT = 6.8 min and RT = 8.1 min; each group contained three compounds with slightly different retention times. (Figure 2). All compounds showed similar UV/VIS spectra, MS/MS fragmentation patterns, and no hits in natural product databases. The first group of compounds at RT = 6.8 min showed [M + H]^+^ molecular ions of *m*/*z* 1081.5, 1082,5, and 1126.5 Da. The compounds were isolated, and their structures were determined by NMR spectroscopy (Figures S11–S33), MS/MS fragmentation (Figure S38–S40), and Marfey’s method to assign the stereochemistry (Figures S34–S37). This resulted in the assignment of the previously reported bosamycins B, C, and D. The second group of compounds eluting at RT = 8.1 min showed [M + H]^+^ molecular ions [M + H]^+^ with *m*/*z* 1063.5, 1064.5, and 1108.4 Da. The compound with *m*/*z* 1108.4 was the only compound that could be isolated to perform structure elucidation.

*S. aurantiacus* LU19075 was cultivated in DNPM, and the metabolites were extracted with butanol. The compound with *m*/*z* 1108.4 was further purified by reversed-phase flash purification and size exclusion chromatography. The final HPLC purification step led to the successful isolation of the compound. The structure was determined by extensive analysis of 1D and 2D NMR (Figures S2–S10).

The precise mass of the compound was determined to be *m*/*z* 1108.4496 ([M + H]^+^), indicating a water loss compared to bosamycin C. The molecular formula was calculated to be C_51_H_66_N_9_O_19_ with 24 degrees of unsaturation; thus, we expected an additional double bond or ring formation. The structure assignment by NMR revealed a peptide highly similar to bosamycin C but with chemical shift differences observed in the edited-HSQC spectra (Figure S10). The differences were narrowed down to the Ser-*β*-CH_2_ signal showing a low field shift and the Gly-CH_2_ signal, which showed the emergence of diastereotopic proton signals. Therefore, we suggested ring formation between serine and glycine through condensation as the final step in the biosynthetic pathway. Due to ambiguous HMBC correlations to prove the connection between serine and glycine, further support was required to confirm the cyclic structure. To obtain further evidence, we analyzed the OH-proton signals after exhaustively freeze-drying the sample. OH-proton signals were observed for all tyrosines (δ_H_ 9.11–9.16), as well as the *β*-OH-Asp (δ_H_ 6.11) signals. The only proton signal missing was from OH-serine, which indicates that serine is condensed with the C-terminal carboxyl group (Figures S5 and S7). Due to structural similarities to bosamycin C and side-chain-to-tail lactonization, the new compound was named depsibosamycin C. The proposed cyclic structure of depsibosamycin C was confirmed by MS/MS analysis (Figure S41). The fragmentation pattern showed a-ion fragments resulting from the ring structure and y-ions with a difference of 18 Da corresponding to water loss. The b-ion fragments b_1_–b_4_ were the same since depsibosamycin C and bosamycin C share the same carboxyl carbamoyl moiety at the *N*-terminus.

**Figure 3:**
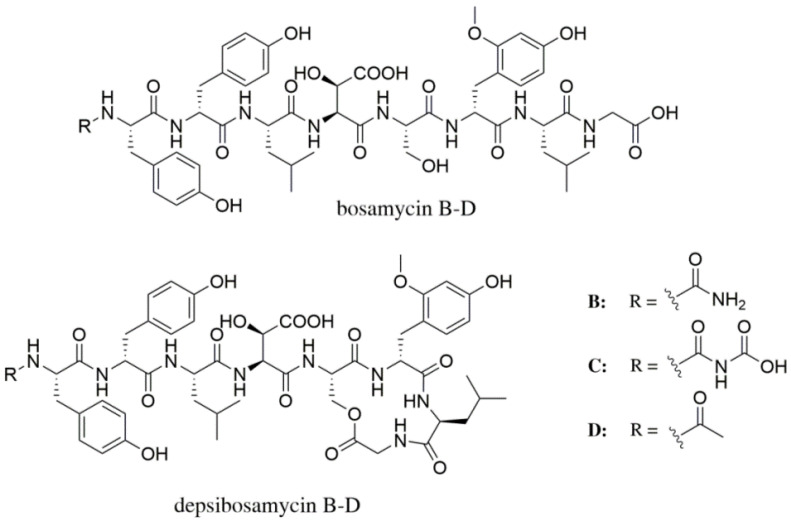
Structures of bosamycins B, C, and D and depsibosamycins B, C, and D.

The other two compounds showed precise [M + H]^+^ molecular ions of *m*/*z* 1064.4598 and 1063.4603 Da and very similar retention times compared to depsibosamycin C. However, the production rate was 20-fold lower; thus, characterisation by MS/MS was used to identify the structures (Figures S42 and S43). The compounds showed the same y-ion pattern as depsibosamycin C, indicating structural similarities. Differences were observed in the b-ion fragments, indicating different *N*-terminal caps. The b-ion fragments b_1_–b_4_ of compound *m*/*z* 1064.5 correspond to those of bosamycin B, while the b-ion fragments b_1_–b_4_ of compound *m*/*z* 1063.5 correspond to those of bosamycin D. In addition, both new compounds are different by 18 Da from their corresponding bosamycins. In conclusion, both new compounds are cyclic variants of linear bosamycins B and D; thus, they were named depsibosamycins B (1064.5) and D (1063.4) (Figure 3).

### 3.2. Heterologous Expression of the Depsibosamycin (dbm) Gene Cluster

**Figure 4:**
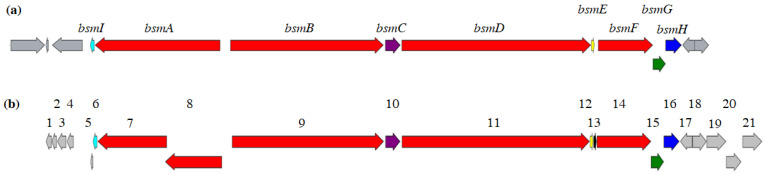
Alignment of the bosamycin (*bsm*) cluster (**a**) and the depsibosamycin (*dbm*) cluster (**b**) revealed 99.2% similarity.

A provided sequenced *S. aurantiacus* LU19075 genomic BAC library was analyzed with the genome mining software antiSMASH in loose settings to identify potential BGC candidates for depsibosamycins. Annotated NRPS clusters in the genome were analyzed in silico regarding their similarity to depsibosamycins by comparing the adenylation domain composition and the stand-alone NRPS module, which is involved in the biosynthesis of 5-OMe tyrosine [19,20,21,22]. One of the NRPS clusters showed an adenylation domain composition that corresponds the depsibosamycin amino acid sequence and the unique stand-alone NRPS unit (*dbmF*), which is expected to produce the 5-OMe tyrosine moiety. Alignment of the *bsm* cluster (GenBank: MN509472) and the identified NRPS cluster from *S. aurantiacus* LU19075, herein called the *dbm* cluster (GenBank: MW740414), showed 99.2% similarity within *dbmI*–*dbmH* (Figure 4). Gene *bsmA* was split into two separate genes, *dbmA*_1_ and *dbmA_2_*; however, the domain organisation remained the same, implying similar function. The *dbm* cluster was chosen as a promising candidate for encoding the biosynthetic pathway of depsibosamycins.

To prove the production of depsibosamycins, the *dbm* cluster was heterologously expressed in *Streptomyces lividans* TK24 [23]. BAC I7, covering the entire *dbm* cluster (Table 1), was identified and isolated from a *S. aurantiacus* LU19075 genomic library.

The BAC was transferred into *Streptomyces lividans* TK24 by biparental conjugation. BAC I7 containing clones were selected from apramycin containing MS plates leading the exconjugant strain *S. lividans* I7. The exconjugants were cultivated in DNPM production medium and extracted with n-butanol. LC-MS revealed successful heterologous expression in *S. lividans* (Figure S47). The production of depsibosamycin D could be readily observed in the extract of the strain, while depsibosamycins B and C were absent. Bosamycin D was produced only in trace amounts. In addition to the known depsibosamycins, two new compounds with *m*/*z* 1021.4523 [M + H] and 1107.4506 [M + H] were identified. Of these two, compound *m*/*z* 1021.4523 [M + H] was produced in the highest amounts. The structures of the new compounds were characterized by MS/MS fragmentation. The y-ion patterns correspond to those previously observed for depsibosamycin B, C, and D. Differences were seen in the b-ion fragments b_1_–b_4_, indicating altered *N*-terminal moieties (Figures S44 and S45). The major compound with *m*/*z* 1021.4523 [M + H] was characterized as depsibosamycin N, the cyclic version of bosamycin N previously discovered by heterologous expression [9]. Comparison of the molecular formula of the compound with *m*/*z* 1107.4506 [M + H] (C_52_H_66_N_8_O_19_) and of depsibosamycin C (C_51_H_66_N_9_O_10_) revealed one more carbon and one fewer nitrogen. The compound did not show similarity to any of the known bosamycins regarding the *N*-terminal cap; hence, it was named depsibosamycin O. The calculated molecular formula of the remaining fragment b_1_ (C_12_H_12_NO_5_^+^) indicates an *N*-malonyl cap at the tyrosine residue (Figure S45).

The spectrum of depsibosamycins produced in the heterologous strain is in accordance with previously published data. Depsibosamycins B and C are two members of the depsibosamycin family carrying carbamoyl and carboxyl carbamoyl *N*-terminal modifications, respectively. As the genes required for the attachment of these moieties are located outside the *dbm* gene cluster, the production of depsibosamycins B and C in heterologous hosts is not expected. Depsibosamycin N is the smallest member of the depsibosamycin family without an *N*-terminal modification. It is produced in the highest amounts, while the modified depsibosamycins D and O are the minor products. The observed depsibosamycins D and O carry acetyl and malonyl moieties at their *N*-termini, respectively. Attachment of the acetyl moiety was proposed to be catalyzed by an unidentified enzyme in the natural bosamycin producer [9]. It is likely that the enzymatic activities required for the attachment of the acetyl and malonyl residues of depsibosamycins D and O are encoded by unidentified genes within the genome of the heterologous host *S. lividans* TK24.

### 3.3. Determination of the Direct Biosynthetic Product of the dbm Gene Cluster

**Figure 5:**
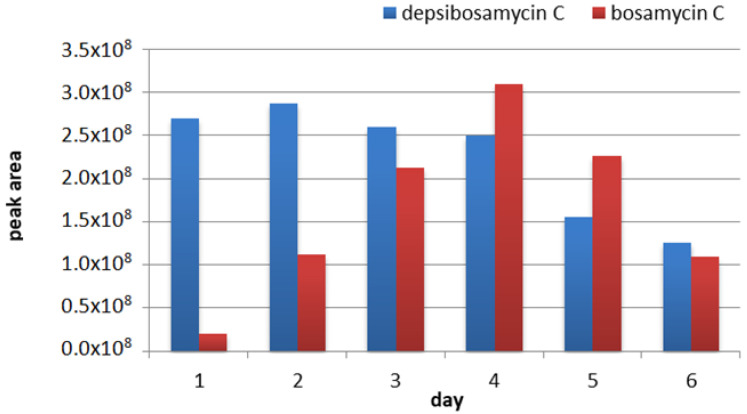
Depsibosamycin C and bosamycin C production in *S. aurantiacus* LU19075 over the course of 6 days.

Both the linear bosamycins and the cyclic depsibosamycins have been identified in the strain *S. aurantiacus* LU19074, while only linear derivatives were reported in a previous study [9]. High similarity of the *bsm* and *dbm* (Figure 4) clusters raises the question of which of the compound families, bosamycins or depsibosamycins, are the direct biosynthetic products. To investigate this, the time course of bosamycin and depsibosamycin production was studied. For this purpose, the *S. aurantiacus* strain was cultivated in DNPM for six days, and the production was screened by LC-MS every day (Figure 5). Cyclic depsibosamycin C was already detected in high amounts after 1 day, while its linear form bosamycin C was barely detectable at this time point. In the course of the following five days, the concentration of depsibosamycin C in the culture did not increase significantly. In contrast, the concentration of bosamycin C increased over the course of cultivation and peaked on the fourth day before it declined again. The obtained data indicate that linear bosamycin C originates from the linearization of cyclic depsibosamycin C. This is in accordance with the heterologous expression results. In the recombinant strain *S. lividans* I7, linear bosamycin D was detected in very low amounts, while cyclic depsibosamycin D was produced in high quantities (Figure S47). Furthermore, depsibosamycins N and O did not appear in their linear forms. The cyclisation of the nascent polypeptide chain is often catalyzed by the thioesterase (TE) domain; thus, we took a closer look at the TE domains of *dbmD* and *bsmD* to determine structural differences. Alterations in the geometry of the TE binding pocket could have catalyzed the formation of the cyclic product by enabling intramolecular esterification [6,7]. However, alignment of the TE domains of *bsmD* and *dbmD* revealed 98% identity (Figure S46), suggesting that altered catalytic activity is unlikely. Furthermore, the sequence analysis of the genes flanking the *dbm* cluster (*orf − 5*–*orf − 1* and *orf + 1*–*orf + 5*) did not reveal any genes that could catalyze the cyclization reaction (Table 1).

This suggests that the TE domain of *DbmD* is responsible for catalyzing the cyclization of the mature linear peptide rather than hydrolytic cleavage. The poor stability of the cyclic depsibosamycins might have prevented their previous discovery in the *Streptomyces sp. 120454* strain.

## 4. Conclusions

In this study, we describe the discovery of the new cyclic depsibosamycins B, C, and D from the strain *S. aurantiacus* LU19075. The compounds consist of the same amino acids as the previously discovered bosamycins B, C, and D but differ by the side-chain-to-tail lactonization of serine and glycine. By in silico analysis, we identified a depsibosamycin (*dbm*) gene cluster. Heterologous expression of the *dbm* cluster revealed the production of cyclic depsibosamycin D and the two new depsibosamycins N and O. Linear bosamycin D was detected only in small quantities. Since the cyclic depsibosamycins have not been reported previously, the origin of cyclic depsibosamycins and linear bosamycins was investigated. By comparing the production rates of linear bosamycin C and cyclic depsibosamycin C, we found that the linear bosamycin likely originates from cyclic depsibosamycin. Furthermore, we compared the two biosynthetic gene clusters, in particular, the TE domains. No significant difference could be detected, suggesting the same product for both gene clusters. Therefore, we postulate that the linear bosamycins might be a product of enzymatic or physicochemical degradation.

## Figures and Tables

**Table 1:** Genes encoded within the chromosomal fragment cloned in BAC I7 and annotation of the *bsm* and *dbm* genes.

Gene #	*dbm* Genes ^a^	Putative Function	*bsm* Genes ^b^
1	*orf − 5*	Hypothetical protein	
2	*orf − 4*	Hypothetical protein	
3	*orf − 3*	Hypothetical protein	
4	*orf − 2*	Hypothetical protein	
5	*orf − 1*	Dehydrogenase	
6	*dbmI*	Acyl carrier protein	*bsmI*
7	*dbmA_1_*	NRPS (C-A-PCP-E)	*bsmA*
8	*dbmA_2_*	NRPS (C-A-PCP)	
9	*dbmB*	NRPS (C-A-PCP-C-A-PCP-C-A-PCP)	*bsmB*
10	*dbmC*	Dioxygenase	*bsmC*
11	*dbmD*	NRPS (C-A-PCP-E-C-A-PCP-C-A-PCP-TE)	*bsmD*
12	*dbmE*	MbtH family protein	*bsmE*
13		Hypothetical protein	
14	*dbmF*	NRPS (P450-A-PCP)	*bsmF*
15	*dbmG*	Alpha/beta hydrolase	*bsmG*
16	*dbmH*	O-methyltransferase	*bsmH*
17	*orf + 1*	ABC transporter	
18	*orf + 2*	ABC transporter	
19	*orf + 3*	Hypothetical protein	
20	*orf + 4*	ABC transporter	
21	*orf + 5*	Hypothetical protein	

^a^ GenBank accession number MW740414 ^b^ GenBank accession number MN509472.

## Data Availability

Not applicable.

## Supplementary Materials

The following are available online at https://www.mdpi.com/article/10.3390/microorganisms9071396/s1, Figure S1: LC-MS spectra of isolated bosamycin B–D and depsibosamycin C, Figures S2–S6: ^1^H, ^13^C, HSQC, COSY, and HMBC NMR data of depsibosamycin C, Figure S7: Extra dry HMBC spectrum of depsibosamycin C, Figures S8 and S9: N-HSQC and ROESY spectrum of depsibosamycin C, Figure S10: Overlapping edited HSQC spectra of depsibosamycin C and bosamycin C, Figures S11–S17: ^1^H, ^13^C, HSQC, COSY, HMBC, N-HSQC, and ROESY NMR data of bosamycin B, Figures S18–S27: ^1^H, ^13^C, HSQC, COSY, HSQC-TOCSY, HMBC, N-HSQC, and ROESY NMR data of bosamycin C, Figures S28–S33: ^1^H, ^13^C, HSQC, COSY, HMBC, and NOESY NMR data of bosamycin D, Figure S34: Marfey’s method: MS spectra of bosamycin C, Figure S35: Structure of bosamycin C with stereochemistry, Figure S36: ^1^H NMR spectrum of DL-*erythro*-*β*-hydroxyaspartic acid, Figure S37: ^1^H NMR spectrum of DL-*threo*-*β*-hydroxyaspartic acid, Figures S38–S40: MS/MS spectra of bosamycin B–D, Figures S41–S43: MS/MS spectra of depsibosamycin B–D, Figure S44: MS/MS spectra of depsibosamycin N, Figure S45: MS/MS spectra of depsibosamycin O, Figure S46: TE domain alignment of the depsibosamycin cluster (Query) and the bosamycin cluster (Sbjct), Figure S47: LC-MS chromatograms of S. lividans I7 with the dbm gene cluster, Table S1: Strains, BACs, plasmids, and primers used in this work, Table S2: NMR data for depsibosamycin C, Table S3: NMR data for bosamycin B, Table S4: NMR data for bosamycin C, Table S5: NMR data for bosamycin D.
