# Supplementary material for: Discovery and Heterologous Production of New Cyclic Depsibosamycins
Source: Microorganisms. 2021 Jun 28;9(7):1396. doi: 10.3390/microorganisms9071396 (PMC8303602; doi:10.3390/microorganisms9071396)
Supplement: Supplementary file 1 [file microorganisms-09-01396-s001.zip › microorganisms-1267553-supplementary.pdf]

microorganisms

MDPI

Discovery and gene cluster of depsibosamycins – cyclic bosamycins from *Streptomyces aurantiacus* LU19075

Marc Stierhof[1], Maksym Myronovskyi[1], Josef Zapp[2] and Andriy Luzhetskyy[1,3,*]

[1] Department of Pharmaceutical Biotechnology, Saarland University, 66123 Saarbruecken, Germany; marc.stierhof@uni-saarland.de (M.S.); maksym.myronovskyi@uni-saarland.de (M.M.)

[2] Department of Pharmaceutical Biology, Saarland University, 66123 Saarbruecken, Germany; j.zapp@mx.uni-saarland.de (J.Z.)

[3] AMEG Department, Helmholtz Institute for Pharmaceutical Research Saarland

[*] Correspondence: a.luzhetskyy@mx.uni-saarland.de; Tel.: +49-681-302-70200 (A.L.)

Supplementary.

1. Strains, BACs, Plasmids and Primers Used in this Work. 4
2. High Resolution Masse of the Isolated Bosamycin B-D and Depsibosamycin C. 5
3. NMR Data of the Isolated Bosamycin B-D and Depsibosamycn C. 6
4. Stereoechemical Assignment by Marfey's Method. 31
5. MS/MS Fragmentation Data. 34

Table of Figures

Figure S1: LC-MS spectra of isolated bosamycin B-D (1-3) and depsibosamycin C (4). 5
Figure S2: ^{1}H NMR spectrum (700 MHz, DMSO-*d6*) of depsibosamycin C.. 8
Figure S3: ^{13}C NMR spectrum (700 MHz, DMSO-*d6*) of depsibosamycin C. 8
Figure S4: Edited HSQC spectrum (700 MHz, 50% NUS, DMSO-*d6*) of depsibosamycin C. 9
Figure S5: Extra dry COSY spectrum (700 MHz, 50% NUS, DMSO-*d6*) of depsibosamycin C showing the serine 28-CH and 28-OH correlation (red circle). 9
Figure S6: HMBC spectrum (700 MHz, 50% NUS, DMSO-*d6*) of depsibosamycin C.............................. 10
Figure S7: Extra dry HMBC spectrum (700 MHz, 50% NUS, DMSO-*d6*/TFA) of depsibosamycin C showing the correlation of 59-NH and C-60 (red circle) and the tyrosine-OH correlations (green circle). 10
Figure S8: N-HSQC spectrum (700 MHz, 25% NUS, DMSO-*d6*/TFA) of depsibosamycin C. 11
Figure S9: ROESY spectrum (700 MHz, DMSO-*d6*/TFA) of depsibosamycin C. 11
Figure S10: Overlapping edited HSQC spectra (700 MHz, DMSO-*d6*/TFA) of depsibosamycin C and bosamycin C.. 12
Figure S11: ^{1}H NMR spectrum (700 MHz, DMSO-*d6*) of bosamycin B.. 15
Figure S12: ^{13}C NMR spectrum (500 MHz, DMSO-*d6*) of bosamycin B. 15
Figure S13: Edited HSQC spectrum (500 MHz, DMSO-*d6*) of bosamycin B. 16
Figure S14: COSY spectrum (700 MHz, DMSO-*d6*) of bosamycin B. 16
Figure S15: HMBC spectrum (700 MHz, DMSO-*d6*) of bosamycin B. 17
Figure S16: N-HSQC spectrum (700 MHz, DMSO-*d6*) of bosamycin B. 17
Figure S17: ROESY spectrum (700 MHz, DMSO-*d6*) of bosamycin B. 18
Figure S18: ^{1}H NMR spectrum (700 MHz, DMSO-*d6*/TFA) of bosamycin C. 21
Figure S19: ^{1}H NMR spectrum (700 MHz, MeOD) of bosamycin C... 21
Figure S20: ^{1}H NMR spectrum (700 MHz, DMSO-*d6*) of bosamycin C. 22
Figure S21: ^{13}C NMR spectrum (700 MHz, DMSO-*d6*) of bosamycin C. 22
Figure S22: HSQC spectrum (700 MHz, DMSO-*d6*) of bosamycin C. 23
Figure S23: COSY spectrum (700 MHz, DMSO-*d6*) of bosamycin C.. 23
Figure S24: HSQC-TOCSY spectrum (700 MHz, DMSO-*d6*) of bosamycin C.. 24
Figure S25: HMBC spectrum (700 MHz, DMSO-*d6*) of bosamycin C. 24
Figure S26: N-HSQC spectrum (700 MHz, DMSO-*d6*) of bosamycin C. 25
Figure S27: ROESY spectrum (700 MHz, DMSO-*d6*) of bosamycin C. 25
Figure S28: ^{1}H NMR spectrum (500 MHz, DMSO-*d6*) of bosamycin D. 28
Figure S29: ^{13}C NMR spectrum (500 MHz, DMSO-*d6*) of bosamycin D. 28

Figure S30: Edited HSQC spectrum (500 MHz, DMSO-*d6*) of bosamycin D. 29
Figure S31: COSY spectrum (500 MHz, DMSO-*d6*) of bosamycin D. 29
Figure S32: HMBC spectrum (500 MHz, DMSO-*d6*) of bosamycin D. 30
Figure S33: NOESY spectrum (500 MHz, DMSO-*d6*) of bosamycin D. 30
Figure S34: Marfey's method: MS spectra of bosamycin C derivatized with D-/L-FDLA (bottom) and the reference amino acids (AA) derivatized with D-/L-FDLA (top). 31
Figure S35: Structure of bosamycin C with stereochemistry determined by Marfey's method.............. 31
Figure S36: ^{1}H NMR spectrum (500 MHz, D_2O) of DL-*erythro*-*β*-hydroxyaspartic acid........................... 32
Figure S37: ^{1}H NMR spectrum (500 MHz, D_2O) of DL-*threo*-*β*-hydroxyaspartic acid............................. 32
Figure S38: MS/MS analysis of bosamycin B. 33
Figure S39: MS/MS analysis of bosamycin C. 33
Figure S40: MS/MS analysis of bosamycin D. 34
Figure S41: MS/MS analysis of depsibosamycin C. 34
Figure S42: MS/MS of depsibosamycin B. 35
Figure S43: MS/MS analysis of depsibosamycin D. 35
Figure S44: MS/MS of depsibosamycin N, produced by *S. lividans* I7. 36
Figure S45: MS/MS analysis and the calculated masses of the expected fragments of depsibosamycin O produced by *S. lividans* I7. 36
Figure S46: TE domain alignment of the depsibosamycin cluster (Query) and the bosamycin cluster (Sbjct). 37
Figure S47: LC-MS chromatograms of *S. lividans* I7 with the dbm gene cluster showing (a) the extracted masses of depsibosamycin D, N and O (b) the extracted mass of bosamycin D, (c) the base peak chromatograms (bpc) of *S. lividans* I7 and (d) the bpc of the empty host *S. lividans* TK24 37

Table of Tables

Table S1: Strains, BACs, plasmids and primers used in this work. 4
Table S2: NMR data (700 MHz, DMSO-*d6*) for depsibosamycin C. 6
Table S3: NMR data (700 MHz, DMSO-*d6*) for bosamycin B. 13
Table S4: NMR data (700 MHz, DMSO-*d6*+TFA) for bosamycin C. 19
Table S5: NMR data (500 MHz, DMSO-*d6*) for bosamycin D. 26

1. Strains, BACs, Plasmids and Primers Used in this Work

Table S1: Strains, BACs, plasmids and primers used in this work.

Material	Purpose
**Bacterial strains**	
Streptomyces *aurantiacus* LU19075	wild type strain [BASF]
Streptomyces lividans TK24	heterologous host[1]
Escherichia coli GB05 RedCC	cloning host [Helmholtz Institute für Pharmaceutische Forschung (HIPS)]
Escherichia coli ET12567 pUB307	alternate host intergeneric conjugation[2]
**BACs**	
I7	Heterologous expression of depsibosamycin cluster
**Plasmids**	
pSMART	Lucigen (USA)

2. High Resolution Masse of the Isolated Bosamycin B-D and Depsibosamycin C

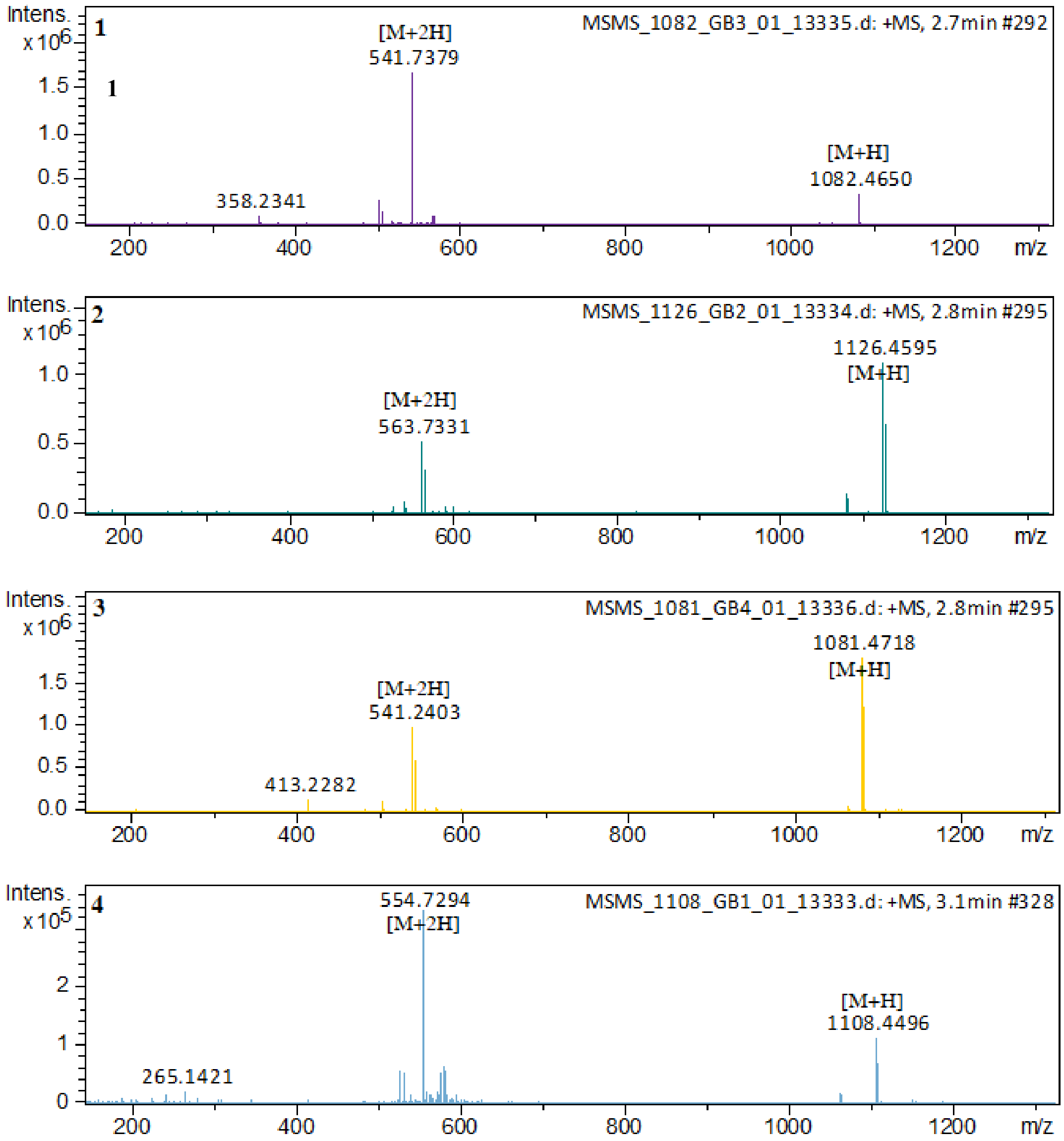

Figure S1: LC-MS spectra of isolated bosamycin B-D (**1-3**) and depsibosamycin C (**4**).

3. NMR Data of the Isolated Bosamycin B-D and Depsibosamycin C

Table S2: NMR data (700 MHz, DMSO-*d6*) for depsibosamycin C.

unit	δ_H, multiplicity, (J in Hz)	δ_C	δ_N
Gly			
1 – COOH		168.7	
2 – $CH_{\alpha,1}$	3.98, dd (16.6, 6.1)	41.6	
$CH_{\alpha,2}$	3.73, m		
3 – NH	7.56, t (6.1)		105.2
Leu			
4 – CO		171.2	
5 – CH_{α}	4.29, m	49.6	
6 – $CH_{\beta,1}$	1.42, ovl*	37.5	
$CH_{\beta,2}$	1.34, m		
7 – CH_{γ}	1.26, m	23.8	
8 – $CH_{\delta,1}$	0.76, d (5.7)	22.8	
9 – $CH_{\delta,2}$	0.70, d (5.5)	21.5	
10 – NH	7.97, d (7.8)		125.5
o-MeO-Tyr			
11 – CO		171.0	
12 – CH_{α}	4.50, ovl*	52.4	
13 – $CH_{\beta,1}$	2.91, m	29.2	
$CH_{\beta,2}$	2.64, ovl*		
14		115.2	
15		158.0	
16	6.33, bs	98.7	
17		157.3	
17 – OH	9.16, bs		
18	6.17, d (7.33)	106.4	
19	6.81, d (7.6)	130.9	
20	3.71, s	55.1	
21 – NH	7.66, d (8.5)		122.8
Ser			
22 – CO		168.5	
23 – CH_{α}	4.52, ovl*	51.8	
24 – $CH_{\beta,1}$	4.11, d (9.9)	65.7	
$CH_{\beta,2}$	4.21, d (8.5)		
25 – NH	8.27, ovl*		116.6
β-OH-Asp			
26 – CO		168.9	
27 – CH_{α}	4.69, t (7.2)	55.2	
28 – CH_{β}	4.16, d (6.6)	70.9	
28 – OH	6.11, bs		
29 – COOH		172.6	
30 – NH	8.19, d (7.5)		111.3

Leu			
31 – CO		172.3	
32 – CH$_{\alpha}$	4.48, ovl*	50.9	
33 – CH$_{\beta}$	1.43, ovl*	41.0	
34 – CH$_{\gamma}$	1.38, m	24.0	
35 – CH$_{\delta,1}$	0.81, ovl*	23.2	
36 – CH$_{\delta,2}$	0.80, ovl*	21.2	
37 – NH	8.26, ovl*		119.8
Tyr			
38 – CO		171.2	
39 – CH$_{\alpha}$	4.48, ovl*	54.7	
40 – CH$_{\beta,1}$	2.84, m	37.3	
CH$_{\beta,2}$	2.67, ovl*		
41		127.2	
42, 46	7.06, d (7.2)	130.2	
43, 45	6.63, d (7.3)	114.8	
44		156.0	
44 – OH	9.11, bs		
47 – NH	8.41, d (6.1)		118.6
Tyr			
48 – CO		170.1	
49 – CH$_{\alpha}$	4.47, ovl*	53.9	
50 – CH$_{\beta,1}$	2.60, ovl*	36.6	
CH$_{\beta,2}$	2.73, m		
51		127.0	
52, 56	6.73, d (7.2)	130.0	
53, 55	6.55, d (7.3)	114.8	
54		155.8	
54 – OH	9.11, bs		
57 – NH	8.26, ovl*		114.7
FCA			
58 – CO		158.7	
59 – NH	11.45, bs		169.2
60 – COOH		156.2	

*signal overlap

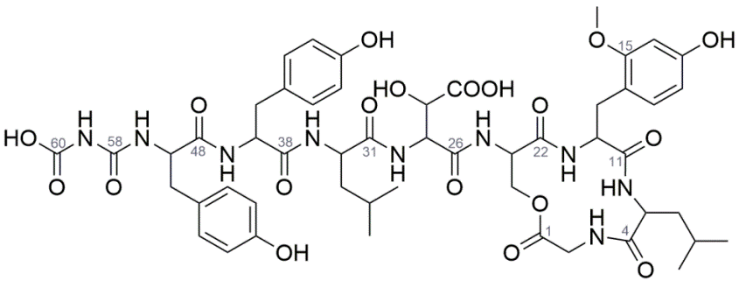

depsibosamycin C

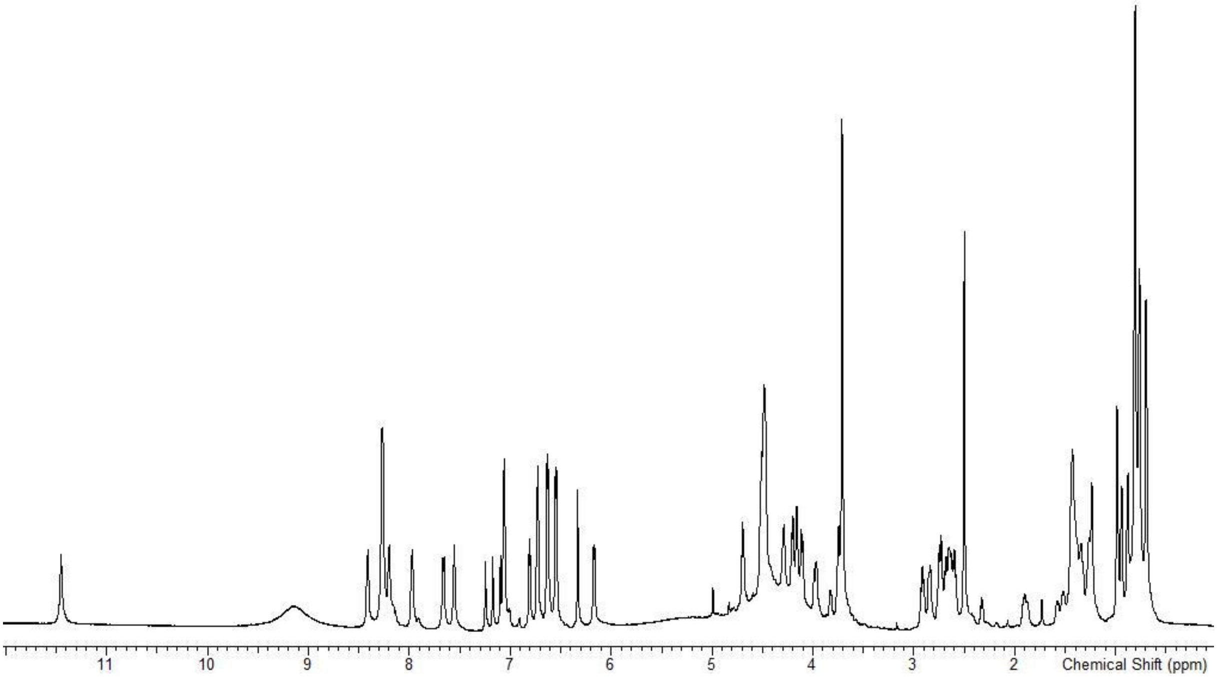

Figure S2: ^{1}H NMR spectrum (700 MHz, DMSO-*d6*) of depsibosamycin C.

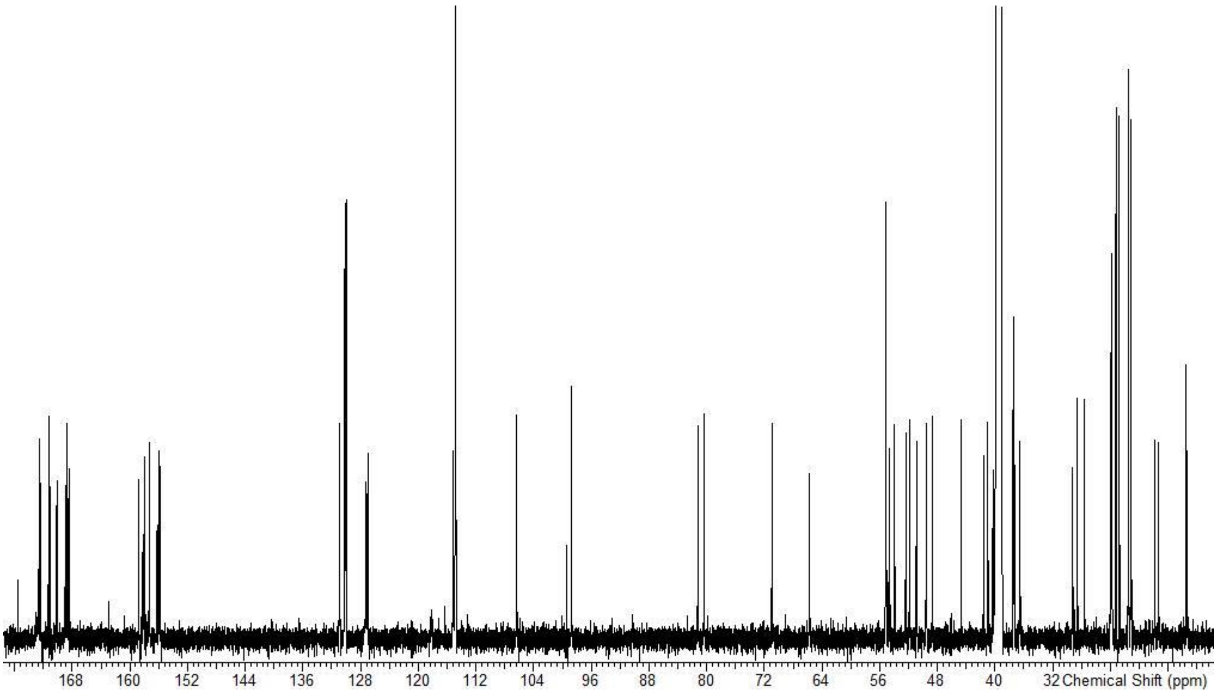

Figure S3: ^{13}C NMR spectrum (700 MHz, DMSO-*d6*) of depsibosamycin C.

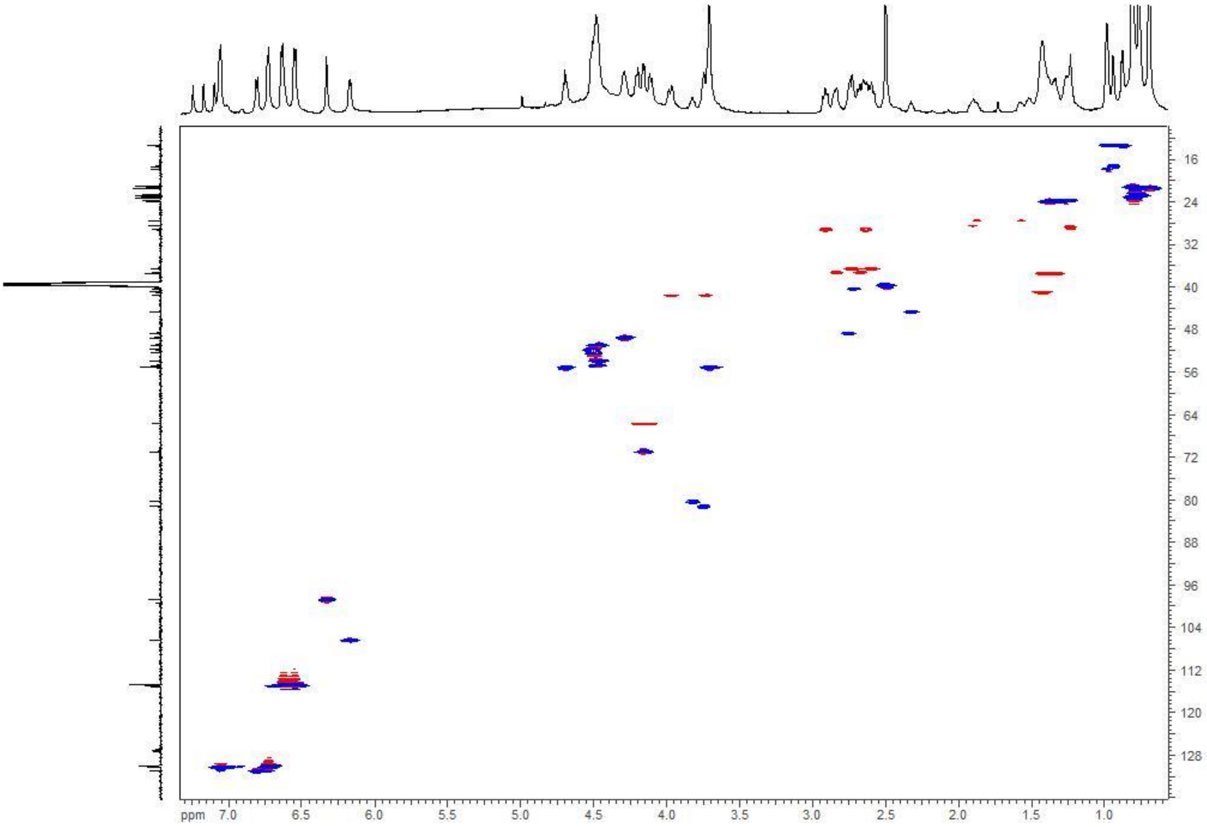

Figure S4: Edited HSQC spectrum (700 MHz, 50% NUS, DMSO-*d6*) of depsibosamycin C.

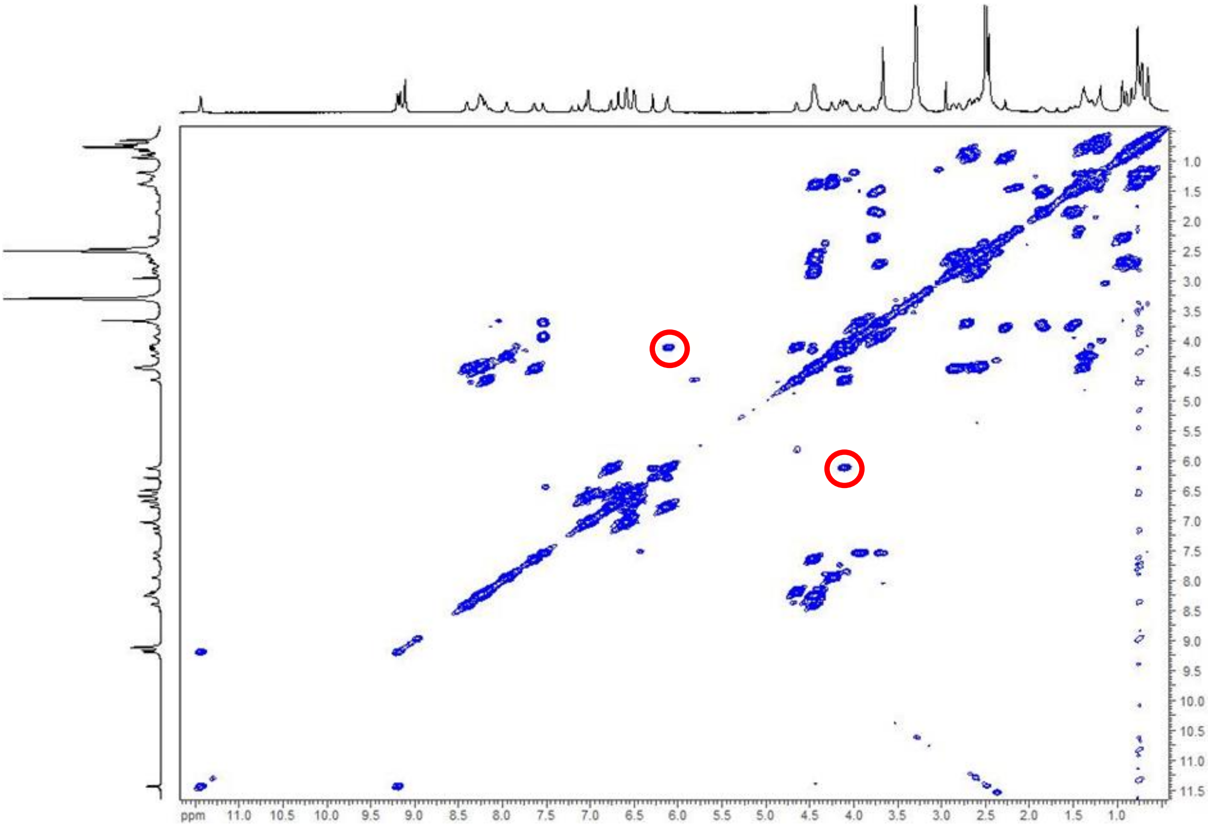

Figure S5: Extra dry COSY spectrum (700 MHz, 50% NUS, DMSO-*d6*) of depsibosamycin C showing the serine 28-CH and 28-OH correlation (red circle).

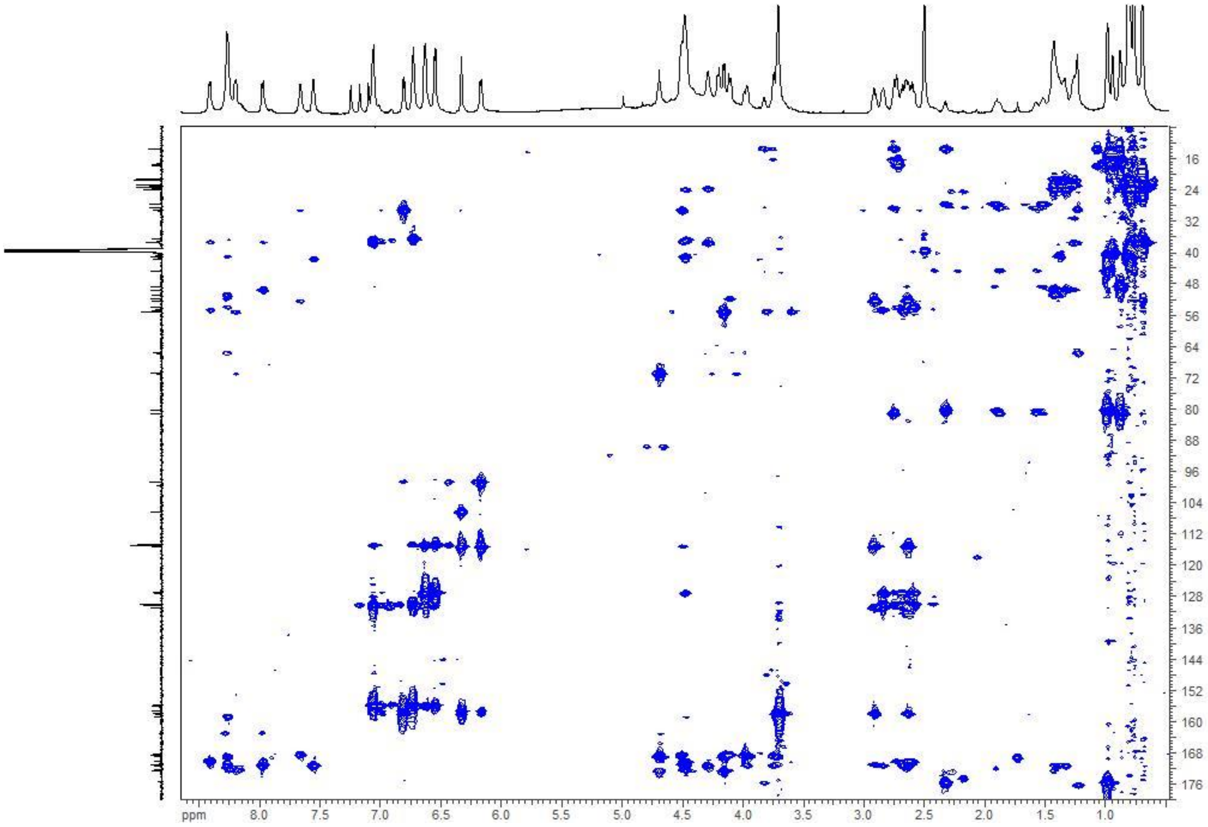

Figure S6: HMBC spectrum (700 MHz, 50% NUS, DMSO-*d6*) of depsibosamycin C.

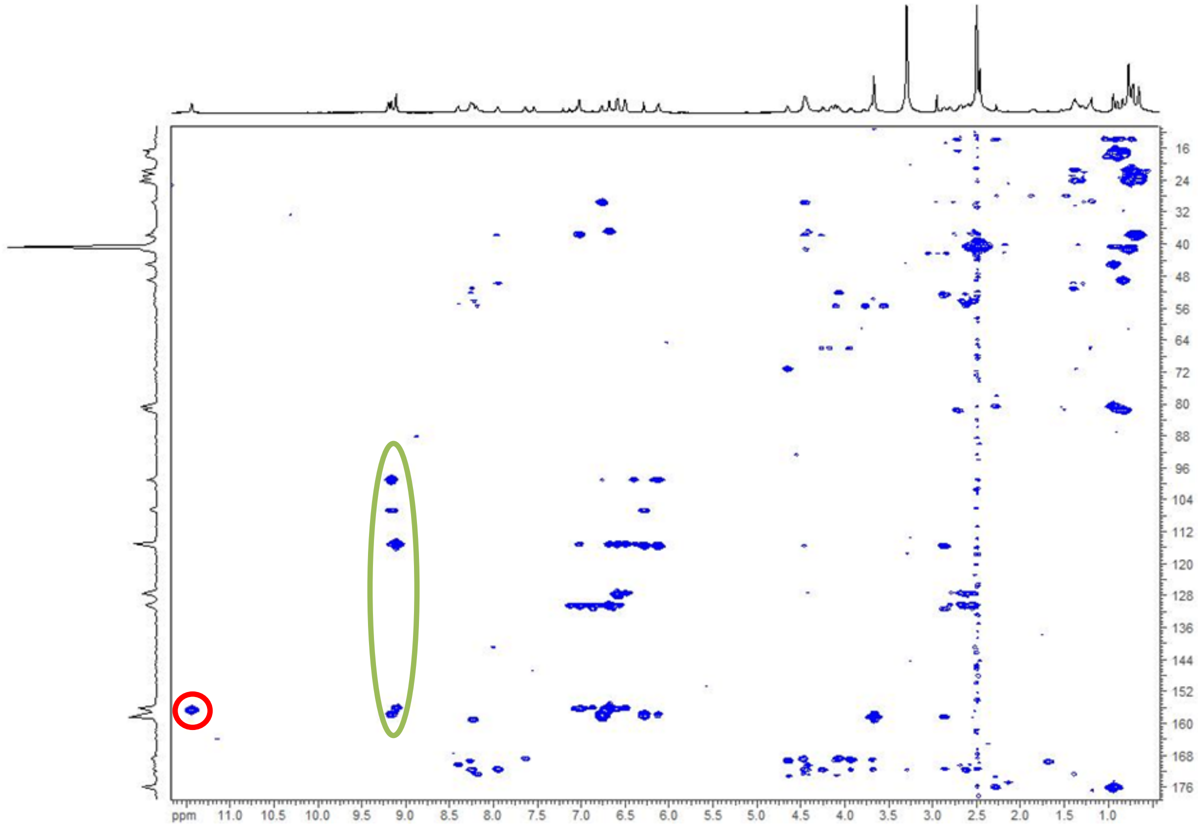

Figure S7: Extra dry HMBC spectrum (700 MHz, 50% NUS, DMSO-*d6*/TFA) of depsibosamycin C showing the correlation of 59-NH and C-60 (red circle) and the tyrosine-OH correlations (green circle).

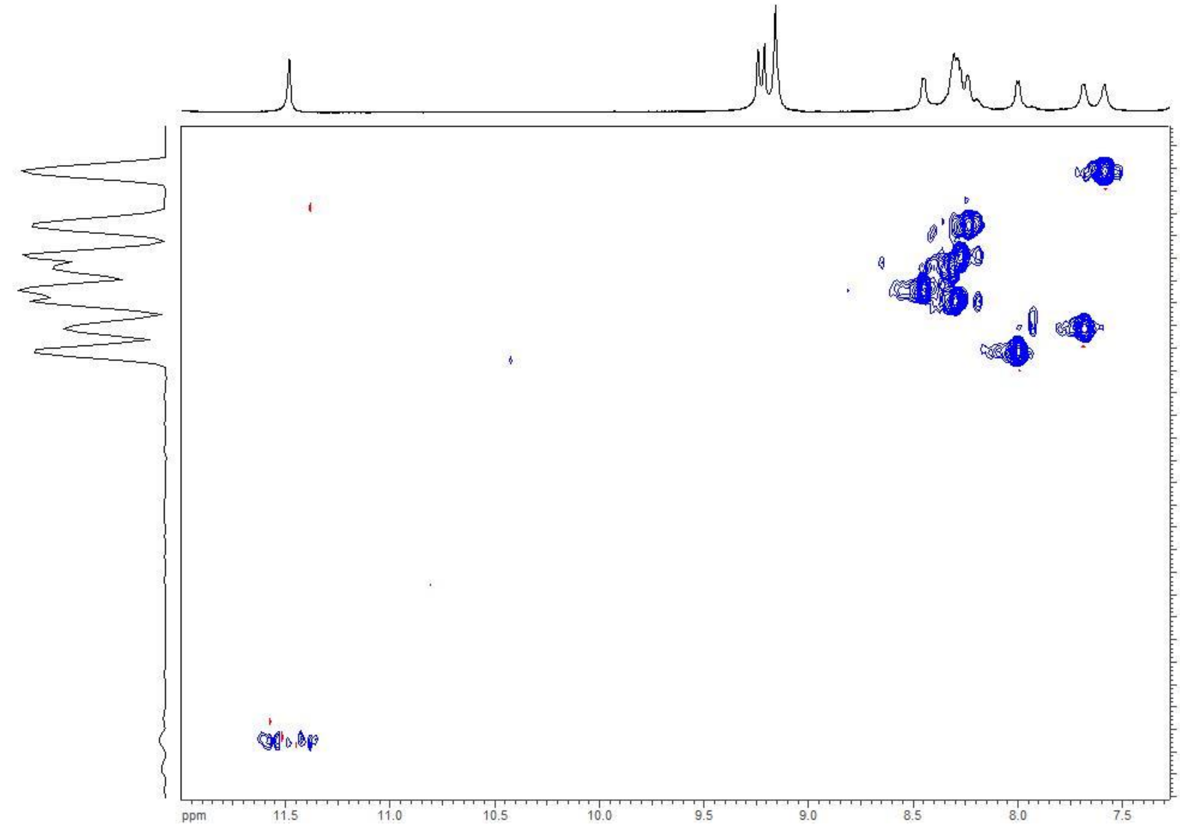

Figure S8: N-HSQC spectrum (700 MHz, 25% NUS, DMSO-*d6*/TFA) of depsibosamycin C.

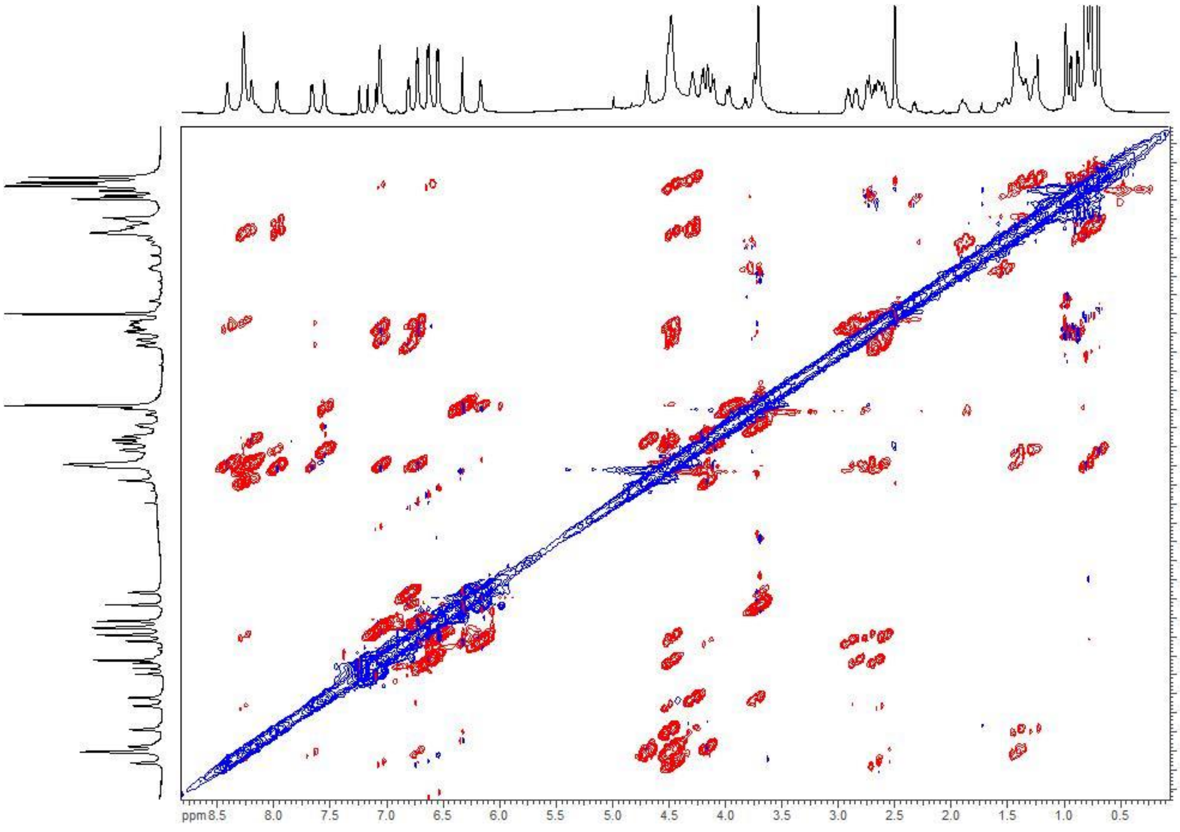

Figure S9: ROESY spectrum (700 MHz, DMSO-*d6*/TFA) of depsibosamycin C.

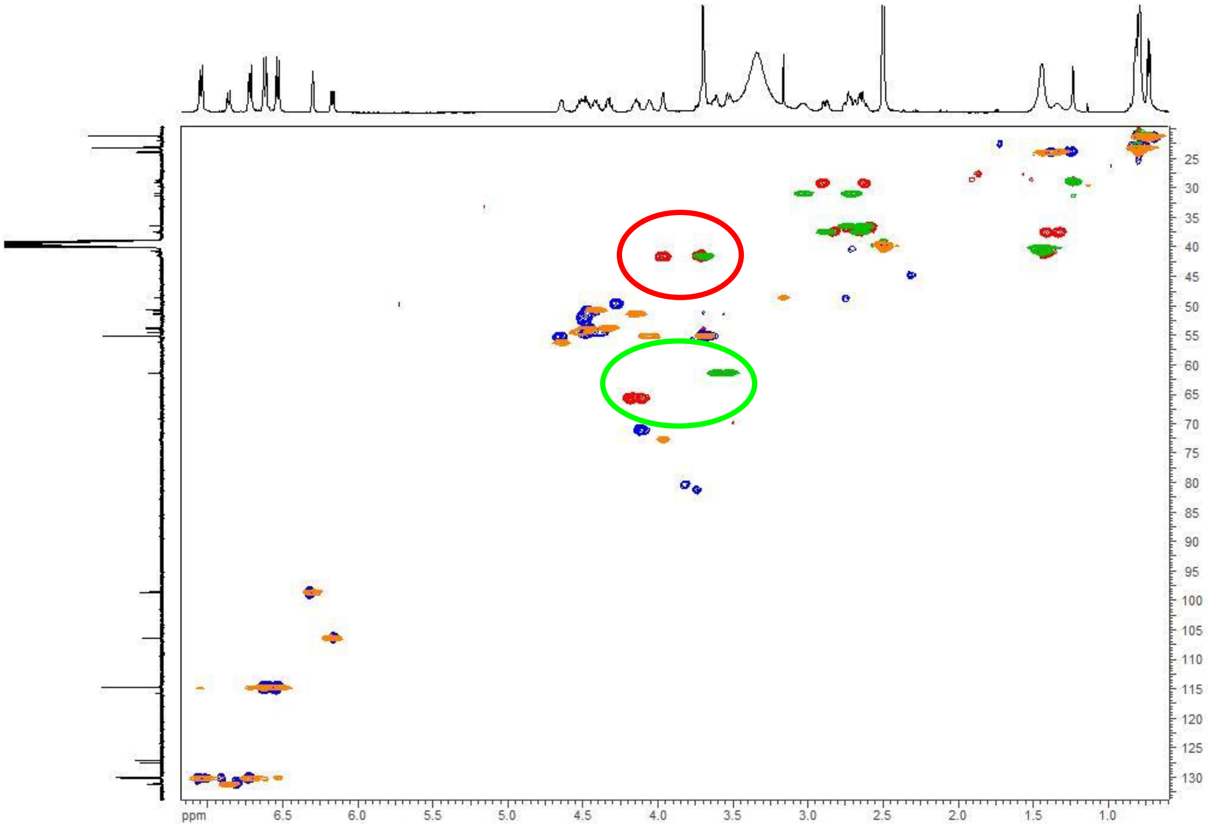

Figure S10: Overlapping edited HSQC spectra (700 MHz, DMSO-*d6*/TFA) of depsibosamycin C and bosamycin C. Methylene/methine signals are depicted as red/blue (dep C) and green/orange (bos C). The methylene signals Gly-CH_2 (red circle) and Ser-CH_β (green circle) of depsibosamycin A showed significant peak splitting (Gly) and a shift difference (Ser) indicating an esterification between Ser-OH and Gly-COOH.

Table S3: NMR data (700 MHz, DMSO-*d6*) for bosamycin B.

unit	δ_H, multiplicity, (J in Hz)	δ_C	δ_N
Gly			
1 – COOH		171.7	
2 – CH_α	3.71, m	41.2	
3 – NH	8.10, t (5.8)		105.2
Leu			
4 – CO		173.2	
5 – CH_α	4.11, m	51.4	
6 – CH_β	1.34, m	40.8	
7 – CH_γ	1.14, m	24.4	
8 – $CH_{\delta,1}$	0.75, d (6.5)	23.7	
9 – $CH_{\delta,2}$	0.68, d (6.5)	22.1	
10 – NH	7.91, d (8.3)		121.3
o-MeO-Tyr			
11 – CO		171.4	
12 – CH_α	4.32, m	54.4	
13 – $CH_{\beta,1}$	2.71, dd (13.5, 7.3)	32.2	
$CH_{\beta,2}$	2.79, dd (13.8, 8.3)		
14		115.5	
15		158.8	
16	6.31, d (2.0)	99.2	
17		158.2	
17 – OH	9.21, bs		
18	6.18, dd (8.1, 2.1)	107.1	
19	6.78, d (8.3)	131.7	
20	3.70, s	55.7	
21 – NH	7.93, d (6.8)		121.3
Ser			
22 – CO		170.5	
23 – CH_α	4.20, m	56.1	
24 – $CH_{\beta,1}$	3.49, ovl*	62.3	
$CH_{\beta,2}$	3.55, dd (10.5, 5.4)		
25 – NH	7.82, d (7.3)		116.3
β-OH-Asp			
26 – CO		169.4	
27 – CH_α	4.72, dd (8.7, 6.0)	55.7	
28 – CH_β	4.08, d (5.8)	71.7	
29 – COOH		173.5	
30 – NH	8.37, d (8.7)		112.5
Leu			
31 – CO		172.9	
32 – CH_α	4.42, m	51.3	
33 – CH_β	1.45, m	41.5	
34 – CH_γ	1.40, m	24.7	

35 – $CH_{\delta,1}$	0.80, d (6.1)	24.1	
36 – $CH_{\delta,2}$	0.78, d (6.1)	21.7	
37 – NH	8.22, d (8.3)		119.5
Tyr			
38 – CO		171.9	
39 – CH_{α}	4.45, m	55.2	
40 – $CH_{\beta,1}$	2.60, dd (13.4, 9.8)	37.9	
$CH_{\beta,2}$	2.85, dd (13.5, 4.4)		
41		128.7	
42, 46	7.07, d (8.5)	131.0	
43, 45	6.62, d (8.5)	115.5	
44		156.6	
44 – OH	9.14, bs		
47 – NH	8.28, d (8.0)		118.5
Tyr			
48 – CO		172.5	
49 – CH_{α}	4.26, m	54.9	
50 – $CH_{\beta,1}$	2.34, dd (13.6, 8.2)	38.3	
$CH_{\beta,2}$	2.55, dd (14.1, 4.0)		
51		128.3	
52, 56	6.67, d (8.3)	130.7	
53, 55	6.55, d (8.3)	115.4	
54		156.2	
54 – OH	9.12, bs		
57 – NH	6.01, d(8.2)		87.2
FCA			
58 – CO		159.0	
59 – NH_2	5.54, bs		76.1

*signal overlapping

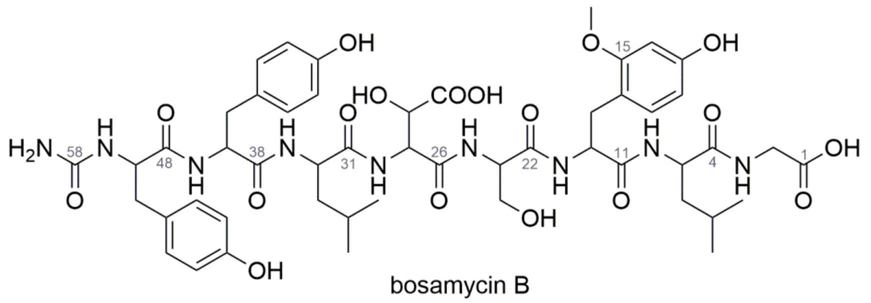

bosamycin B

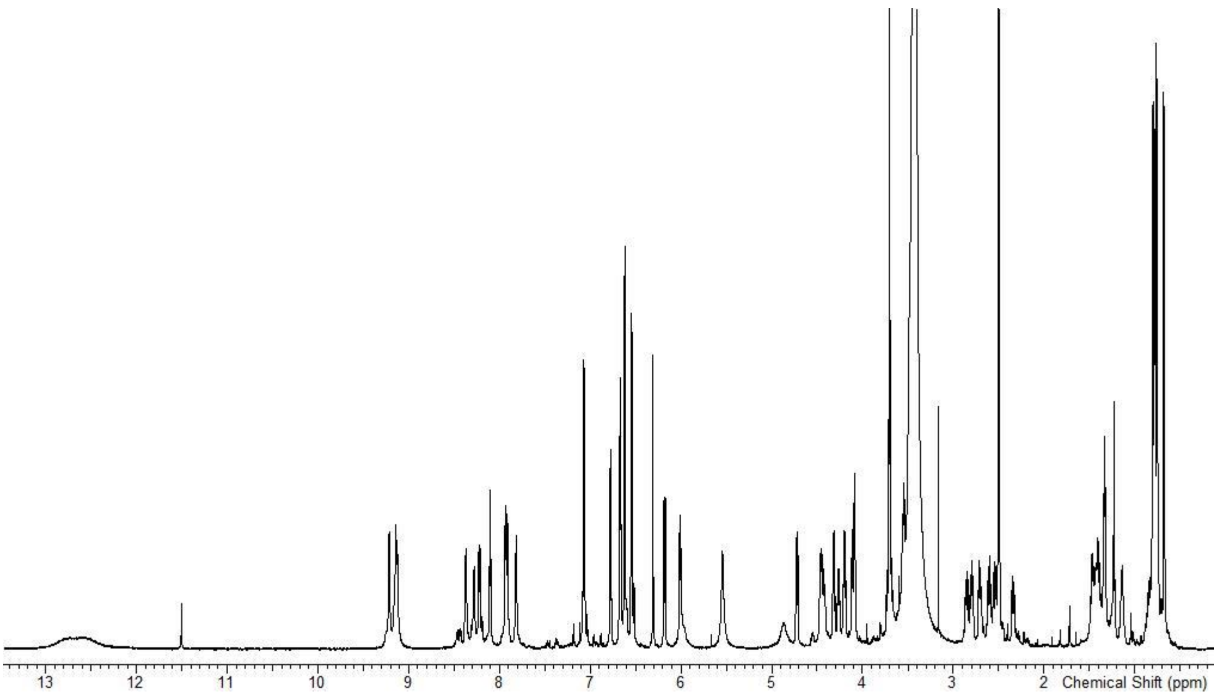

Figure S11: ^{1}H NMR spectrum (700 MHz, DMSO-*d6*) of bosamycin B.

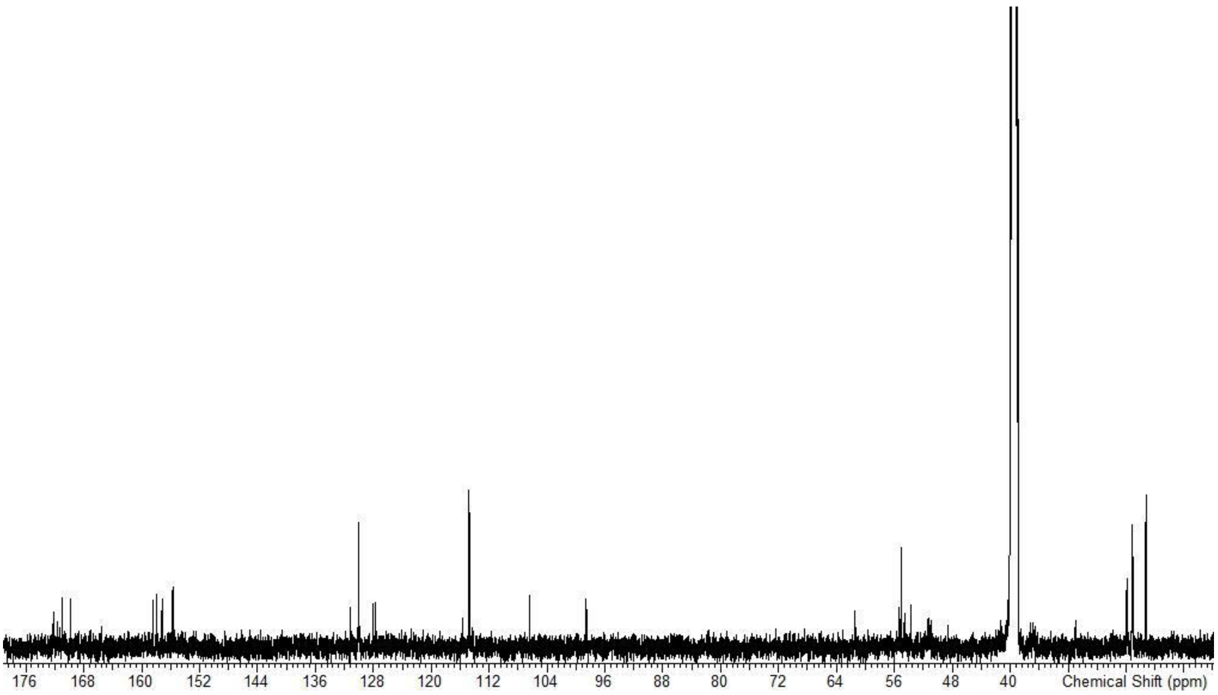

Figure S12: ^{13}C NMR spectrum (500 MHz, DMSO-*d6*) of bosamycin B.

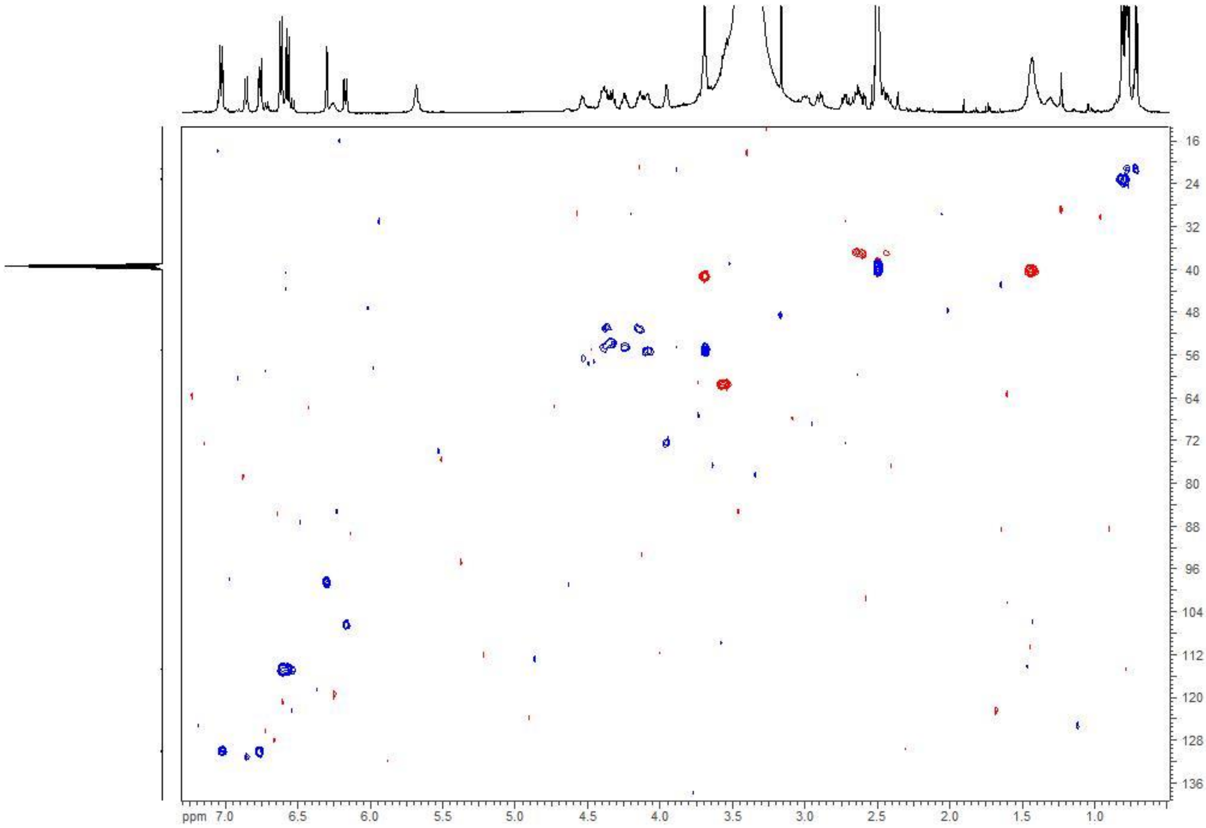

Figure S13: Edited HSQC spectrum (500 MHz, DMSO-*d6*) of bosamycin B.

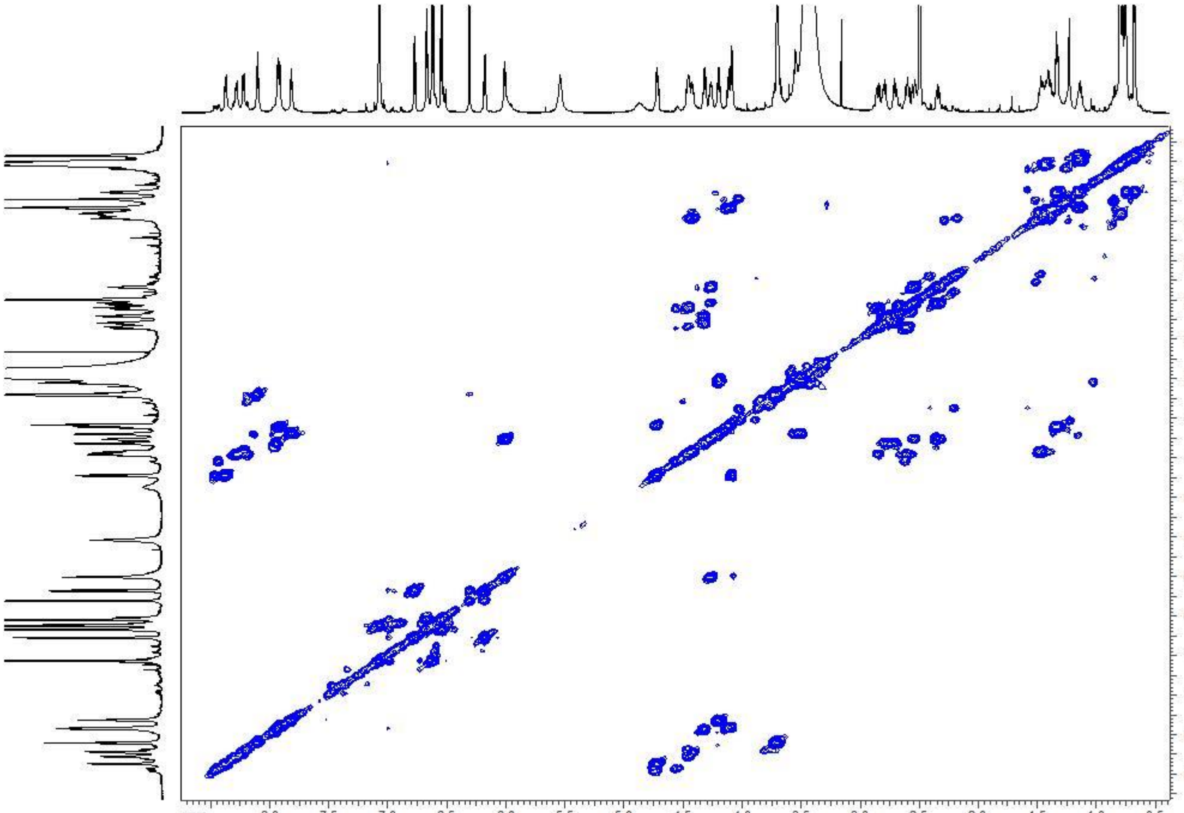

Figure S14: COSY spectrum (700 MHz, DMSO-*d6*) of bosamycin B.

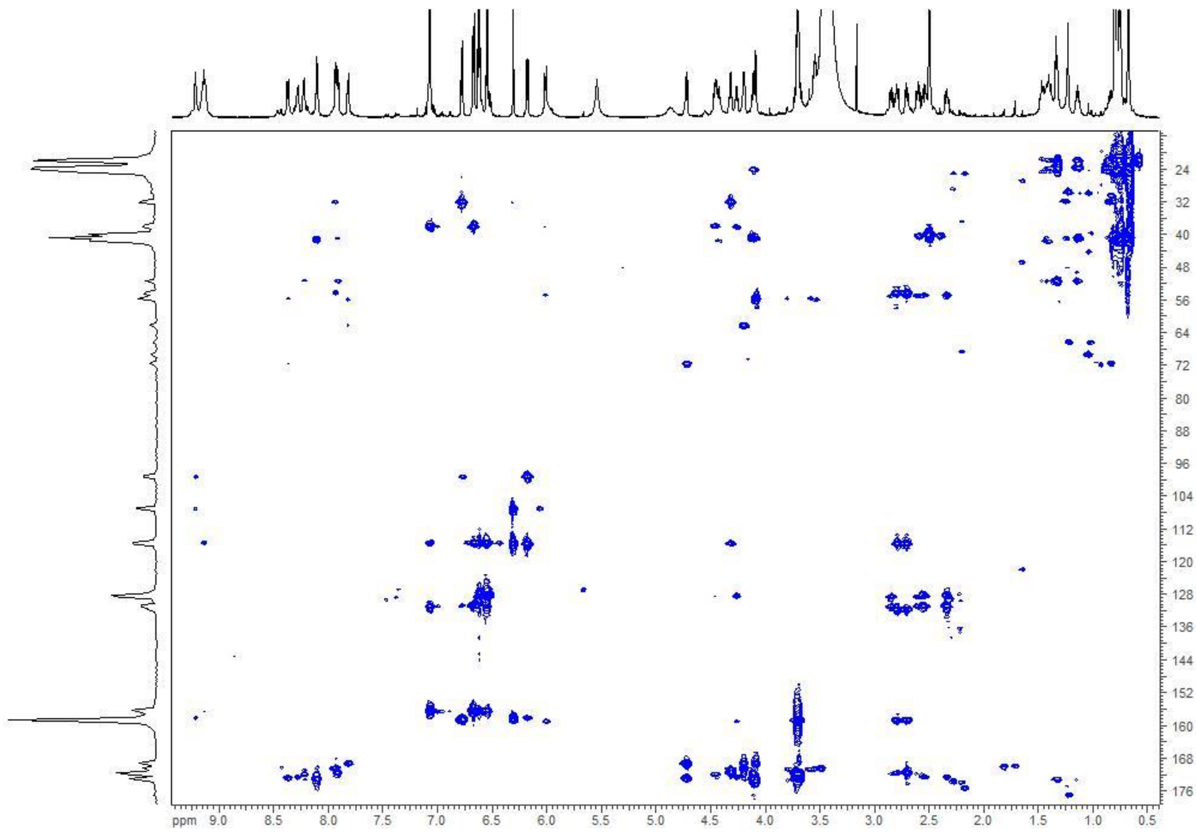

Figure S15: HMBC spectrum (700 MHz, DMSO-*d6*) of bosamycin B.

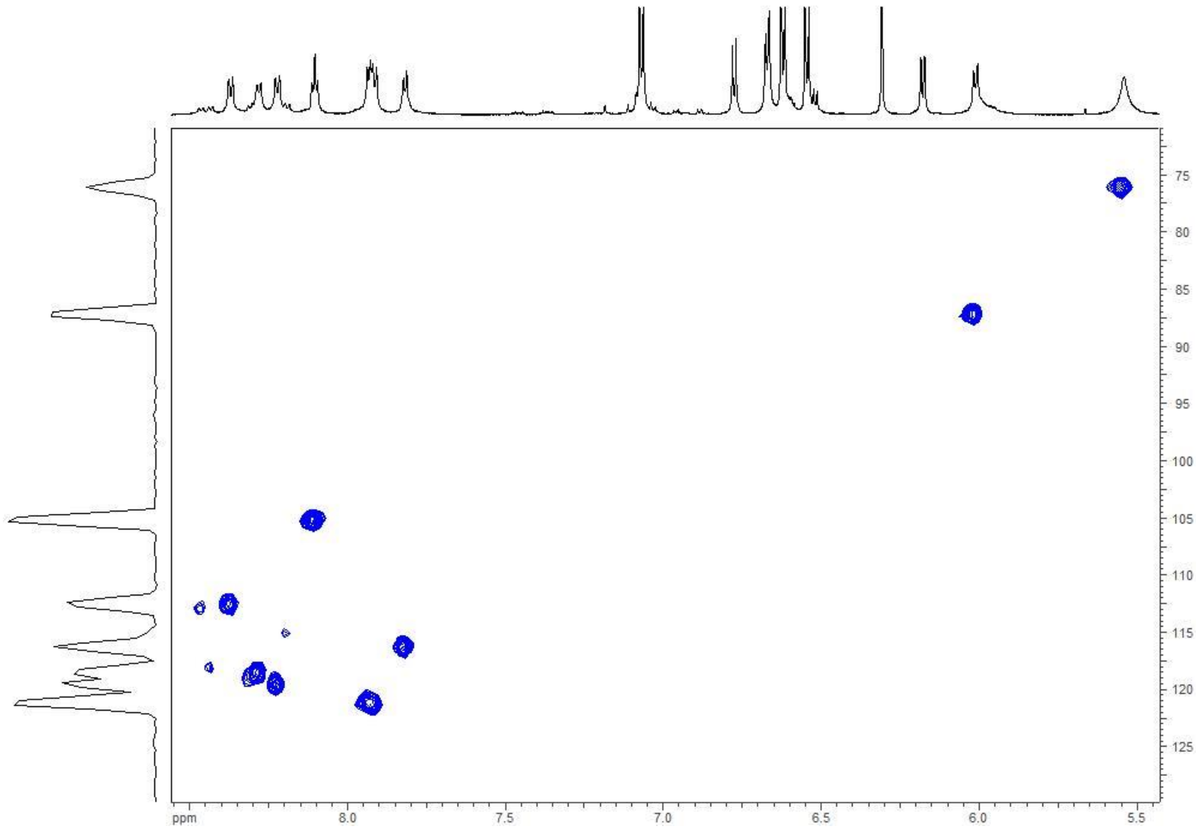

Figure S16: N-HSQC spectrum (700 MHz, DMSO-*d6*) of bosamycin B.

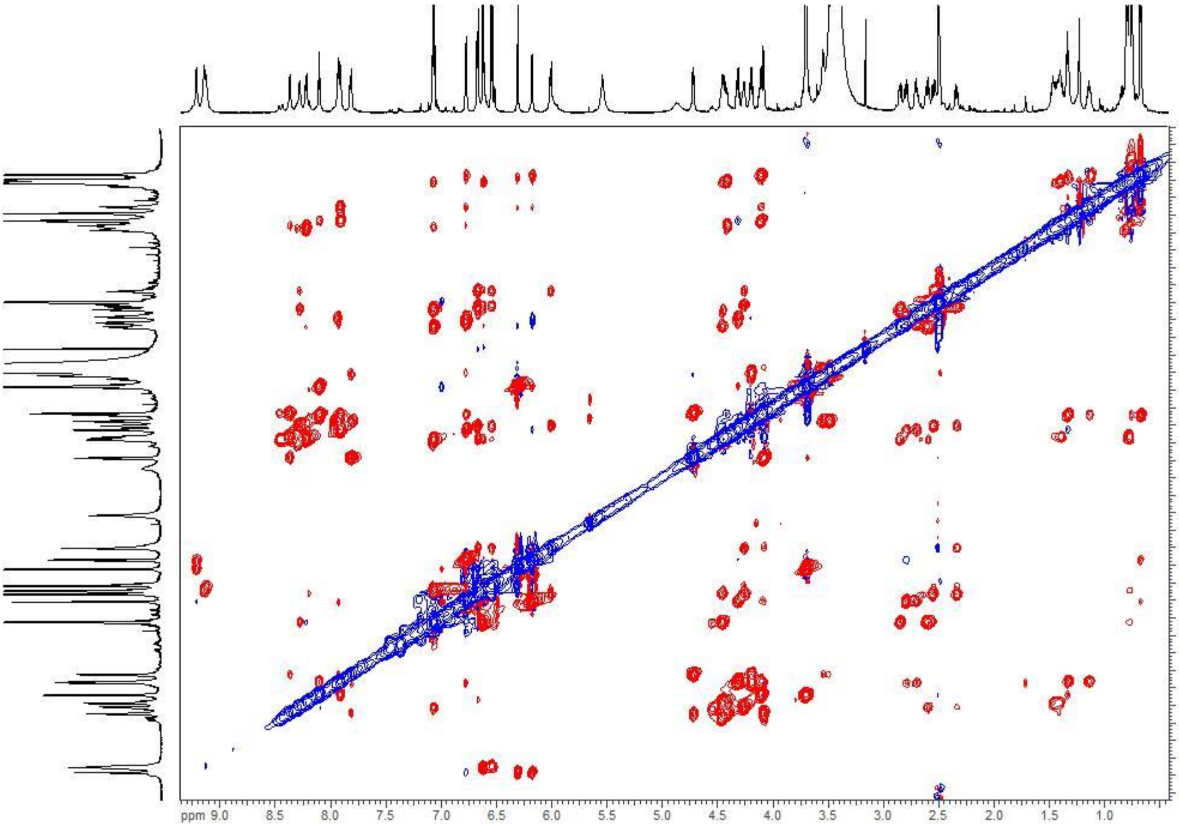

Figure S17: ROESY spectrum (700 MHz, DMSO-*d6*) of bosamycin B.

Table S4: NMR data (700 MHz, DMSO-*d6*+TFA) for bosamycin C.

unit	δ_H, multiplicity, (J in Hz)	δ_C	δ_N
Gly			
1 – COOH		171.4	
2 – CH_α	3.70, ovl*	41.5	
3 – NH	8.53, bs		105.0
Leu			
4 – CO		172.4	
5 – CH_α	4.15, dt (5.2, 9,2)	51.4	
6 – CH_β	1.44, m	40.1	
7 – CH_γ	1.34, m	23.8	
8 – $CH_{\delta,1}$	0.80, ovl*	23.1	
9 – $CH_{\delta,2}$	0.73, d (6.4)	21.2	
10 – NH	7.90, d (8.5)		120.4
o-MeO-Tyr			
11 – CO		171.0	
12 – CH_α	4.33, pq	53.7	
13 – $CH_{\beta,1}$	3.03, m	31.0	
$CH_{\beta,2}$	2.72, ovl*		
14		115.8	
15		158.0	
16	6.30, d (2.2)	98.6	
17		157.2	
17 – OH	9.15, bs		
18	6.17, dd (2.2, 8.1)	106.4	
19	6.87, d (8.2)	131.2	
20	3.70, s	55.1	
21 – NH	8.15, bs		120.4
Ser			
22 – CO		169.9	
23 – CH_α	4.05, m	55.2	
24 – $CH_{\beta,1}$	3.62, dd (4.4, 10.2)	61.4	
$CH_{\beta,2}$	3.52, dd (3.7, 10.5)		
25 – NH	7.68, d (6.5)		115.5
β-OH-Asp			
26 – CO		168.9	
27 – CH_α	4.64, dd (3.6, 7.5)	56.2	
28 – CH_β	3.97, d (3.0)	72.7	
29 – COOH		174.5	
30 – NH	8.11, bs		112.6
Leu			
31 – CO		171.8	
32 – CH_α	4.42, m	50.7	
33 – CH_β	1.44, m	40.8	

34 – CH$_{\gamma}$	1.43, ovl*	24.0	
35 – CH$_{\delta,1}$	0.82, d (5.9)	23.3	
36 – CH$_{\delta,2}$	0.78, ovl*	21.2	
37 – NH	8.23, d (7.9)		119.3
Tyr			
38 – CO		170.7	
39 – CH$_{\alpha}$	4.52, dt (5.1, 8.9)	54.5	
40 – CH$_{\beta,1}$	2.88, dd (5.3, 13.8)	37.5	
CH$_{\beta,2}$	2.66, ovl*		
41		127.6	
42, 46	7.05 (8.4)	130.2	
43, 45	6.62, d (8.4)	114.8	
44		155.9	
44 – OH	9.11, bs		
47 – NH	8.42, d(7.34)		117.9
Tyr			
48 – CO		169.7	
49 – CH$_{\alpha}$	4.48, dt (4.5, 8.7)	54.0	
50 – CH$_{\beta,1}$	2.73, ovl*	36.5	
CH$_{\beta,2}$	2.64, ovl*		
51		127.2	
52, 56	6.72, d (8.4)	130.1	
53, 55	6.54, d (8.4)	114.8	
54		155.8	
54 – OH	9.11, bs		
57 – NH	8.27, d (8.7)		114.9
FCA			
58 – CO		158.8	
59 – NH	11.51, s		168.2
60 – COOH		156.3	

*signal overlapping

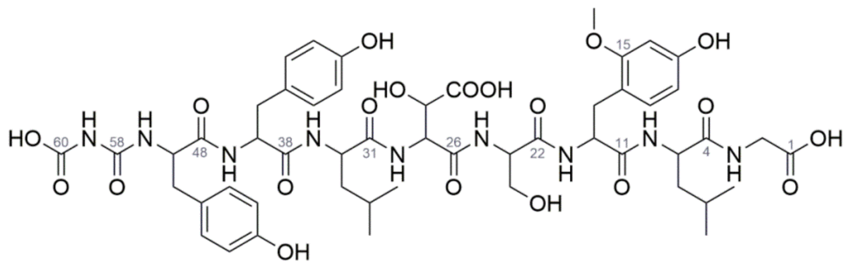

bosamycin C

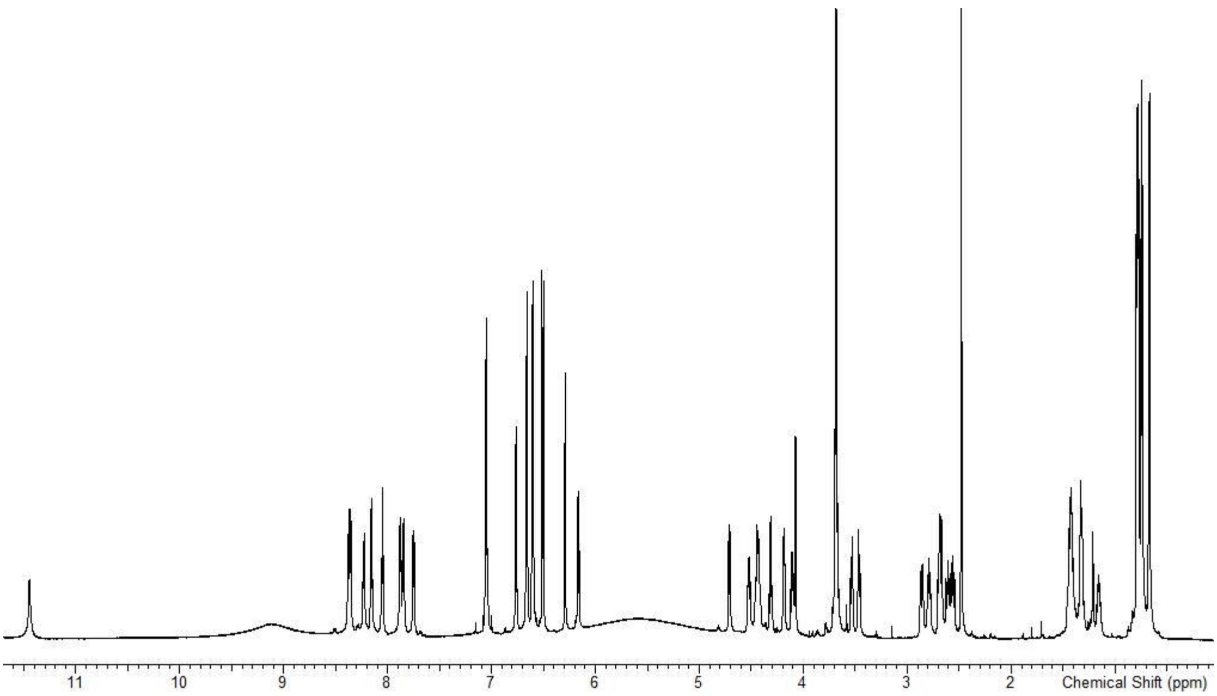

Figure S18: ^{1}H NMR spectrum (700 MHz, DMSO-*d6*/TFA) of bosamycin C.

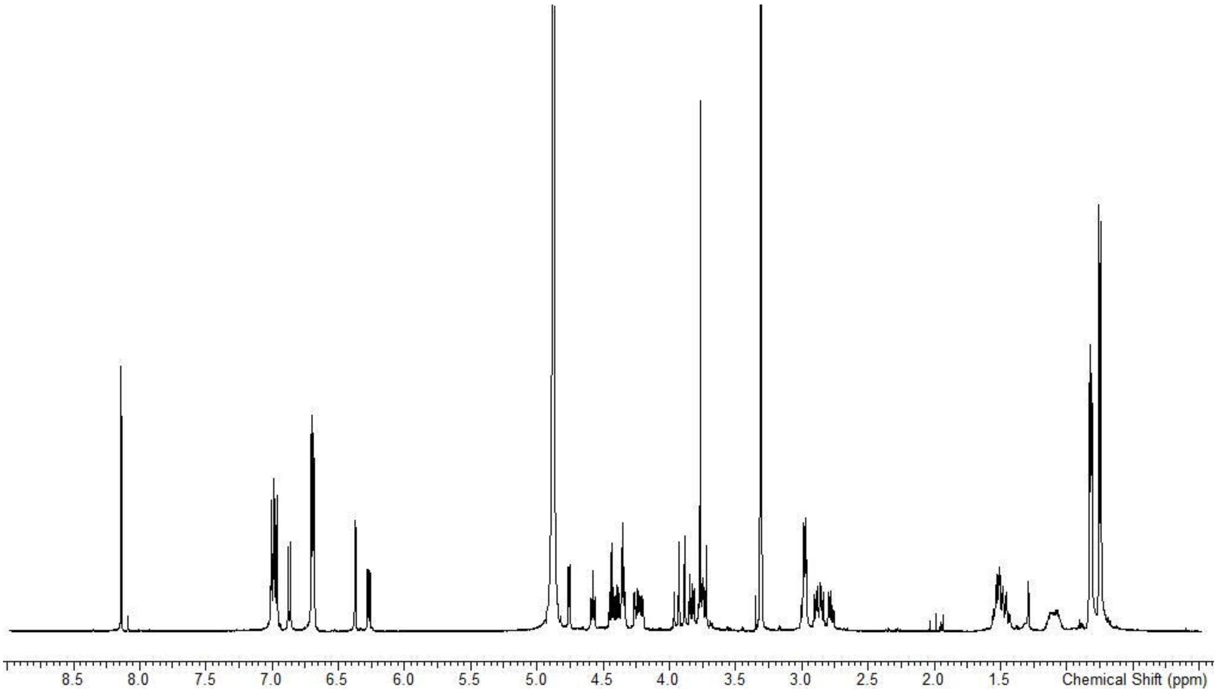

Figure S19: ^{1}H NMR spectrum (700 MHz, MeOD) of bosamycin C.

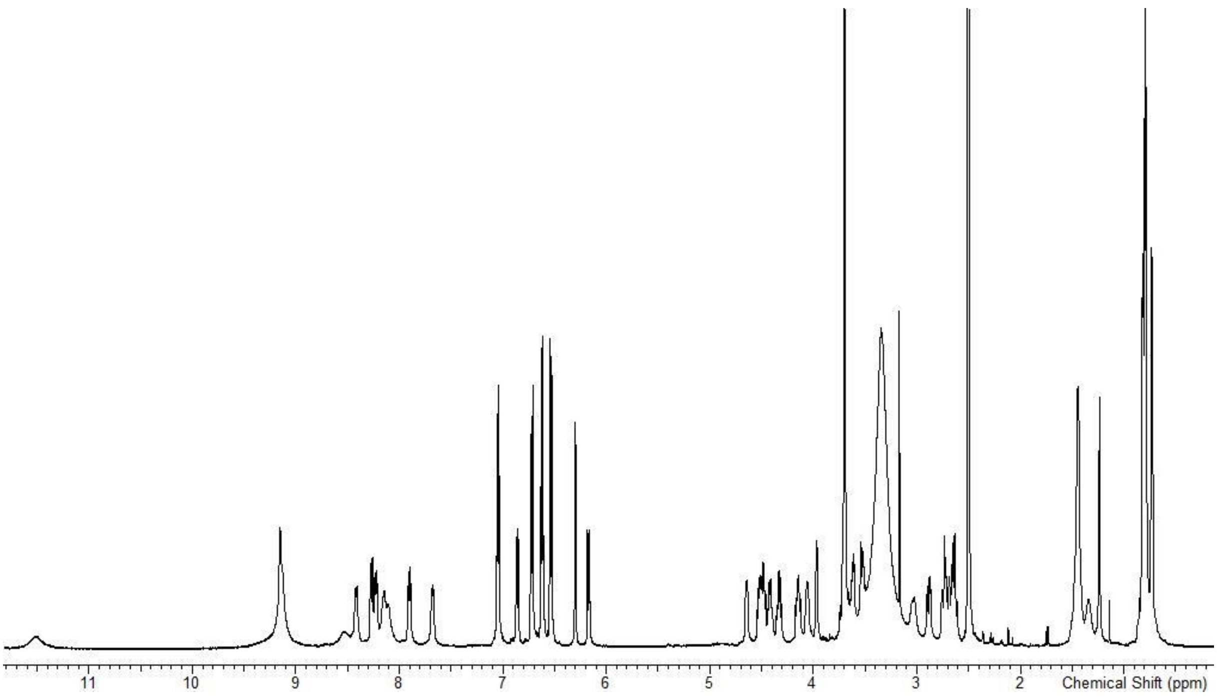

Figure S20: ^{1}H NMR spectrum (700 MHz, DMSO-*d6*) of bosamycin C.

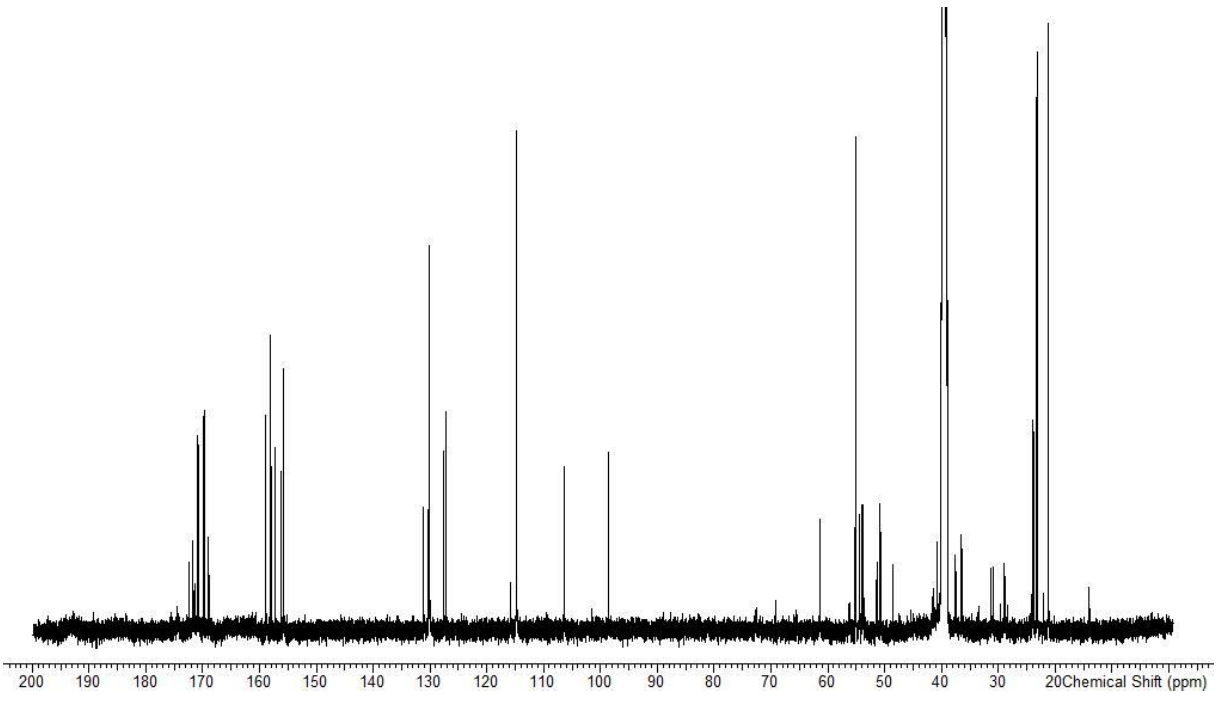

Figure S21: ^{13}C NMR spectrum (700 MHz, DMSO-*d6*) of bosamycin C.

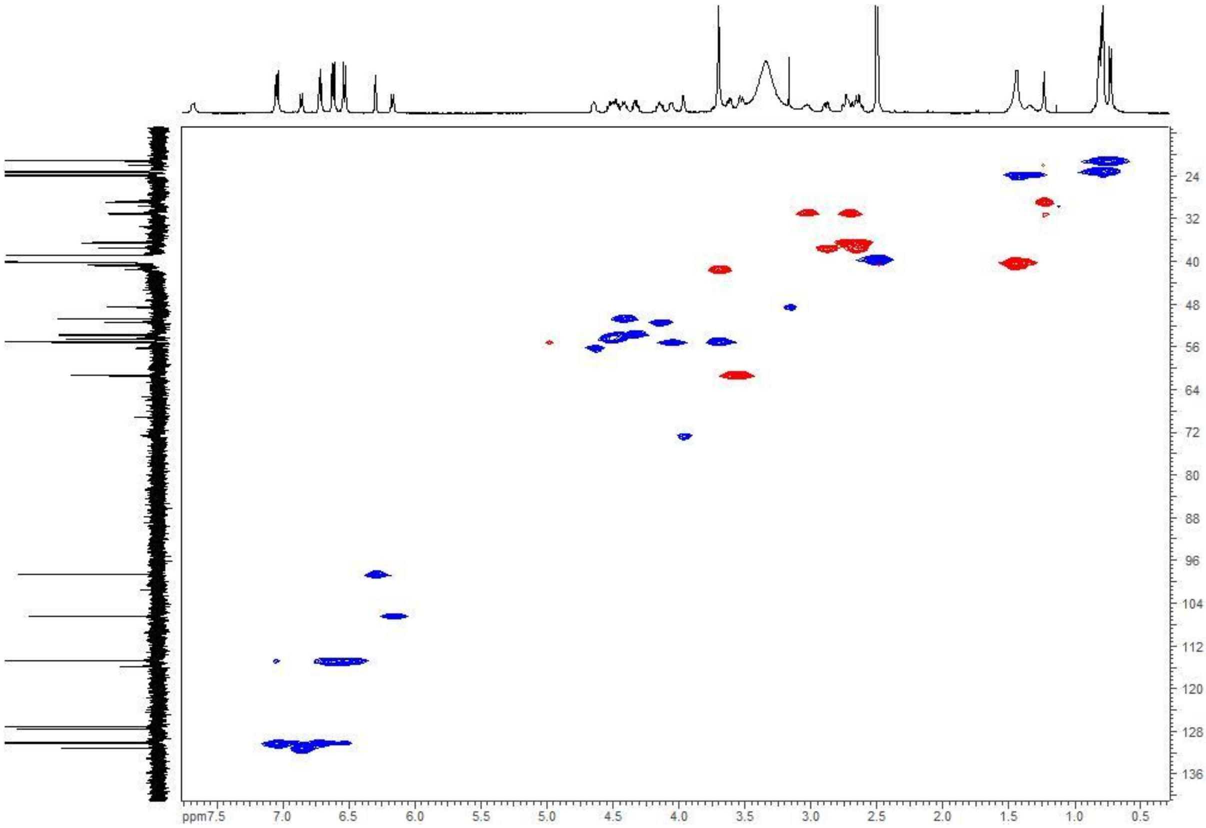

Figure S22: HSQC spectrum (700 MHz, DMSO-*d6*) of bosamycin C.

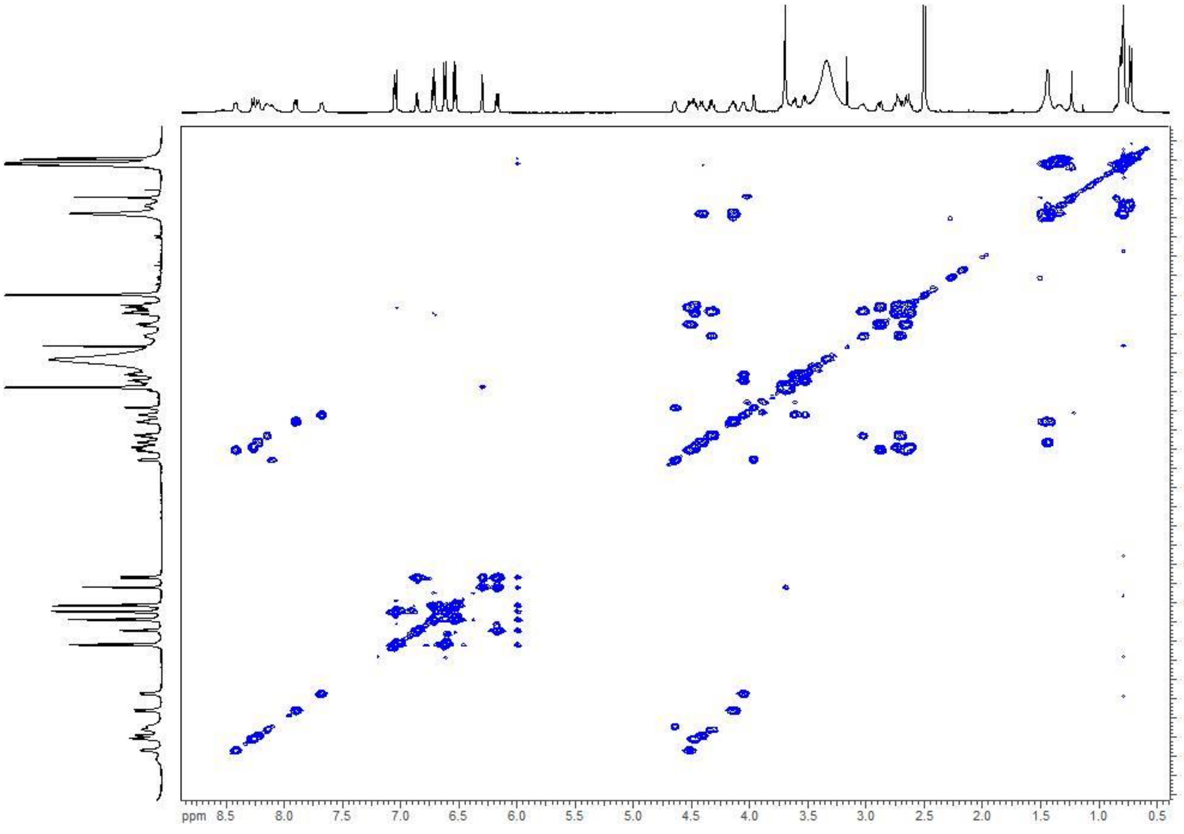

Figure S23: COSY spectrum (700 MHz, DMSO-*d6*) of bosamycin C.

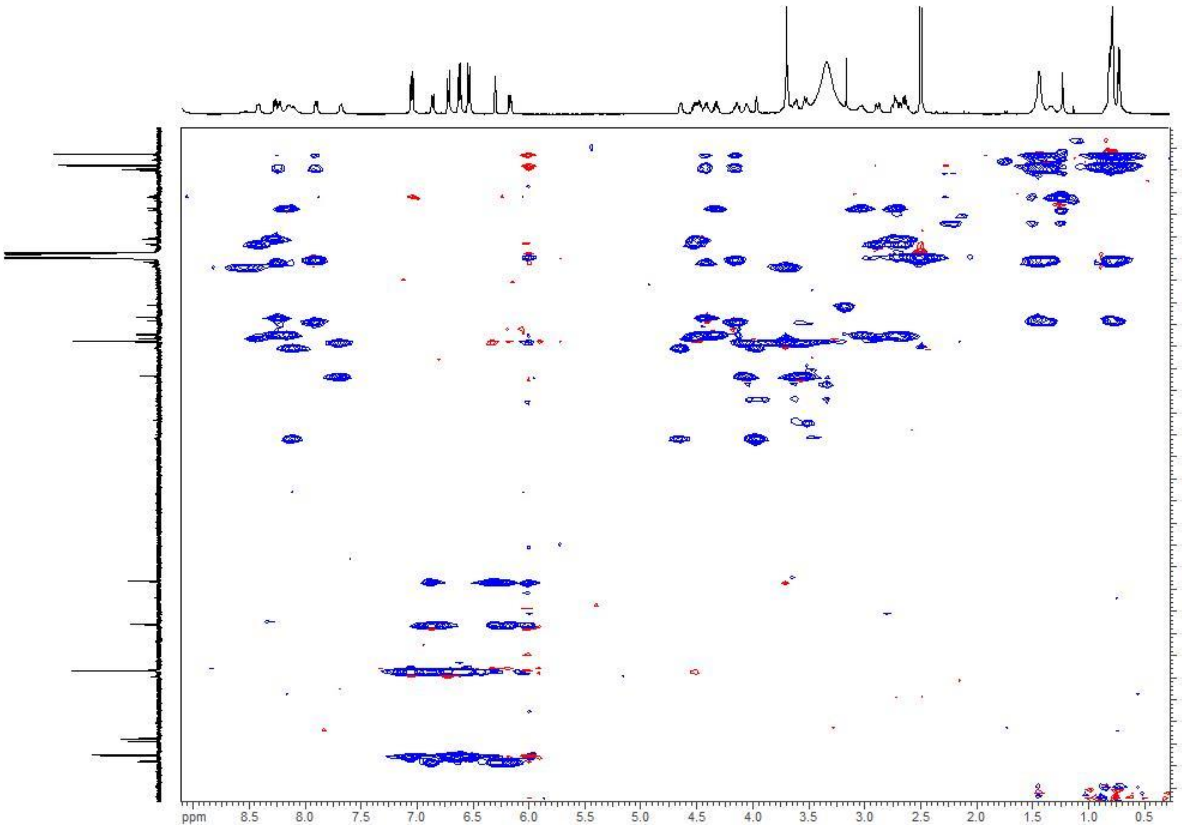

Figure S24: HSQC-TOCSY spectrum (700 MHz, DMSO-*d6*) of bosamycin C.

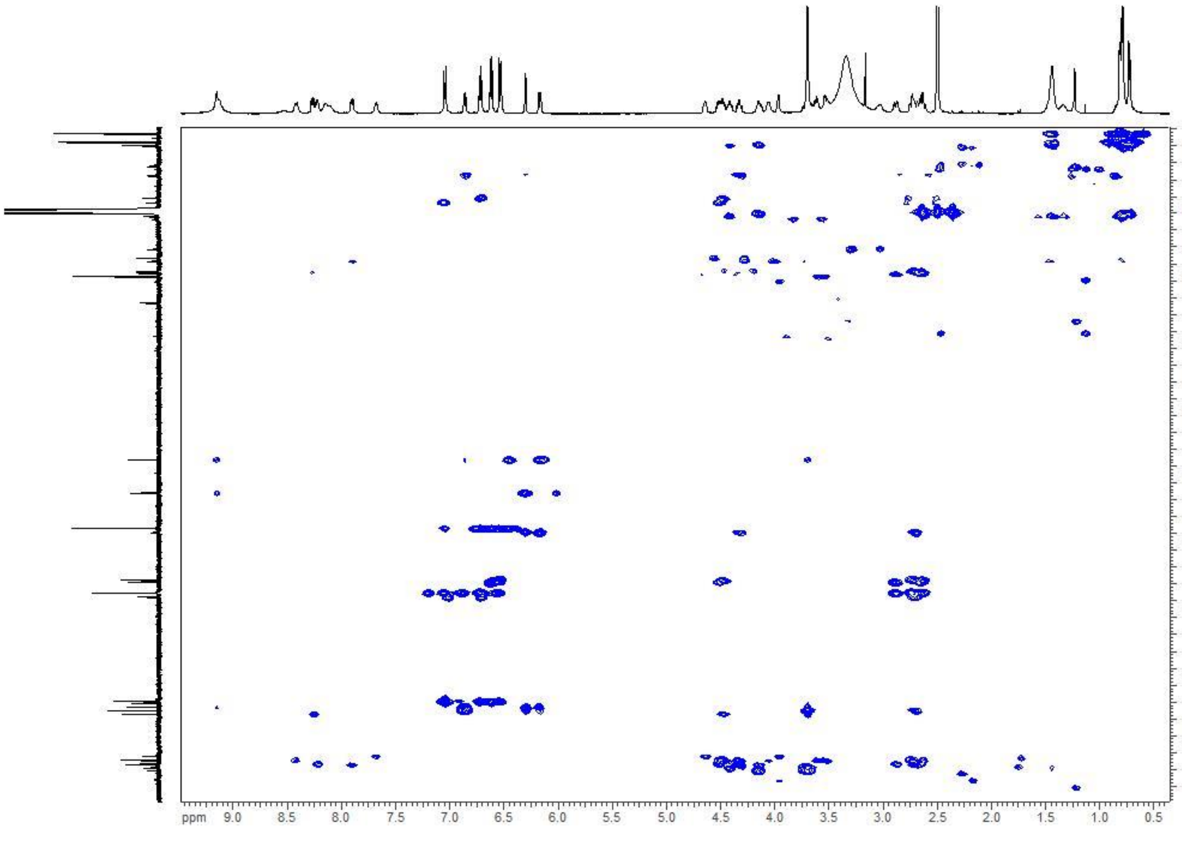

Figure S25: HMBC spectrum (700 MHz, DMSO-*d6*) of bosamycin C.

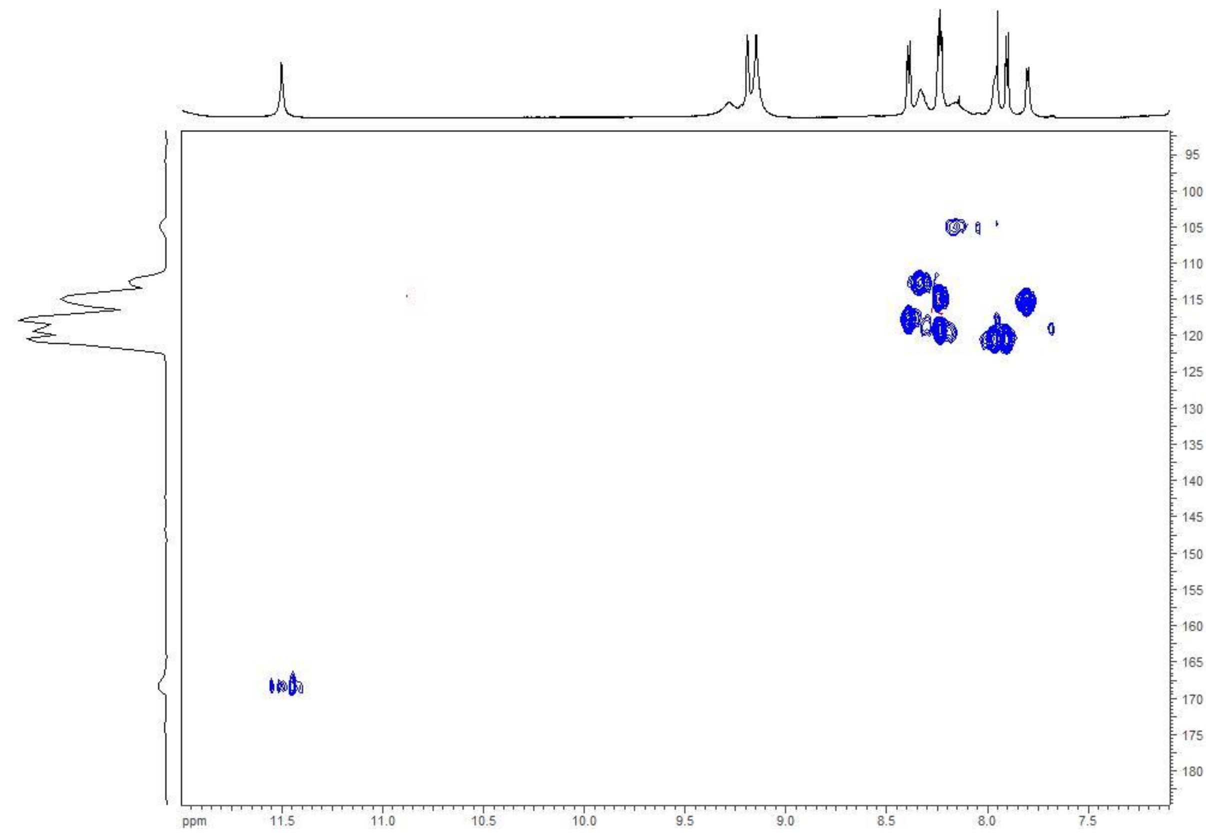

Figure S26: N-HSQC spectrum (700 MHz, DMSO-*d6*) of bosamycin C.

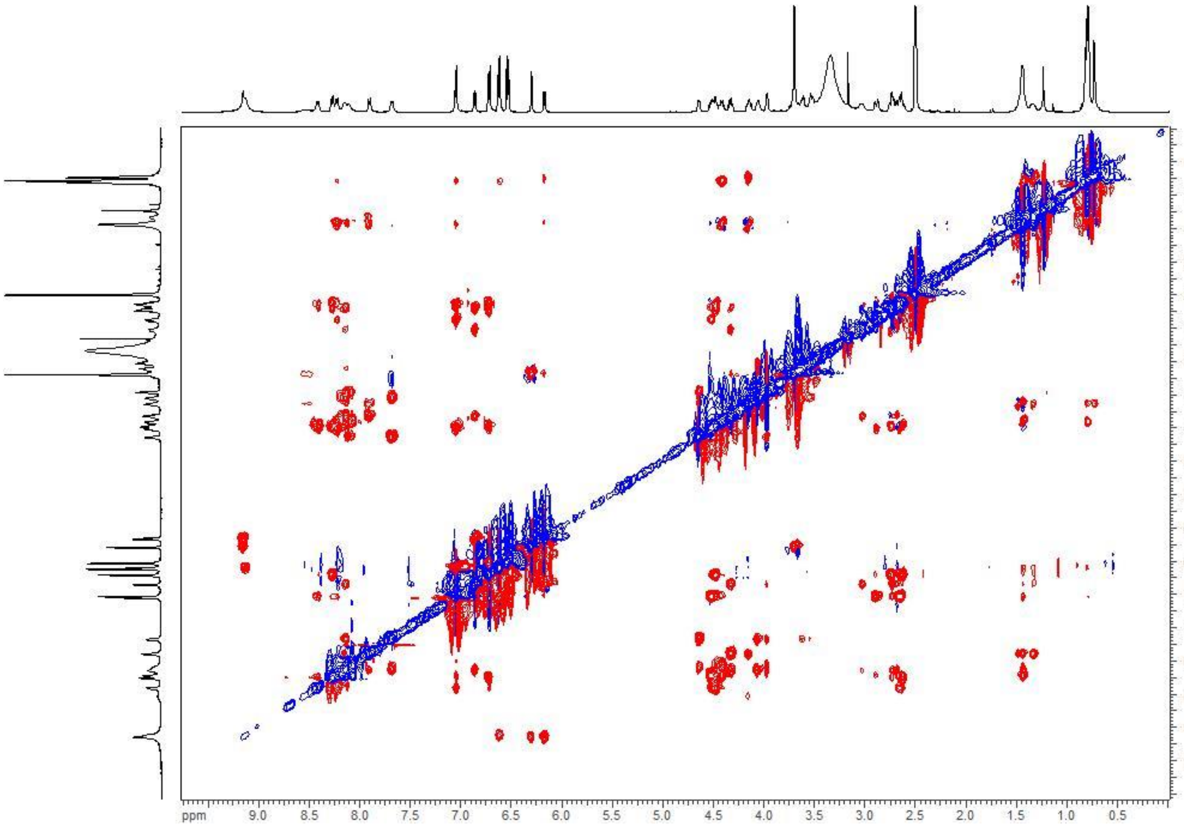

Figure S27: ROESY spectrum (700 MHz, DMSO-*d6*) of bosamycin C.

Table S5: NMR data (500 MHz, DMSO-*d6*) for bosamycin D.

unit	δ_H, multiplicity, (J in Hz)	δ_C
Gly		
1 – COOH		171.1
2 – CH_α	3.71, ovl*	40.8
3 – NH	8.17, ovl*	
Leu		
4 – CO		172.4
5 – CH_α	4.12, ovl*	50.9
6 – CH_β	1.36, t (7.3)	40.3
7 – CH_γ	1.20, m	23.7
8 – $CH_{\delta,1}$	0.76, d (5.0)	23.2
9 – $CH_{\delta,2}$	0.69, d (6.5)	21.3
10 – NH	7.90, d (8.2)	
o-MeO-Tyr		
11 – CO		170.9
12 – CH_α	4.32, dt (7.2, 7.6)	53.7
13 – $CH_{\beta,1}$	2.84, ovl*	31.4
$CH_{\beta,2}$	2.71, dd (13.2, 7.5)	
14		115.2
15		158.1
16	6.31, d (2.2)	98.6
17	-	157.4
17 – OH	9.18, bs	
18	6.18, dd (8.0, 2.12)	106.4
19	6.80, d (8.2)	131.0
20	3.70, s	55.1
21 – NH	7.96, bs	
Ser		
22 – CO		169.8
23 – CH_α	4.16, ovl*	55.3
24 – $CH_{\beta,1}$	3.57, dd (11.0, 5.6)	61.6
$CH_{\beta,2}$	3.51 dd (10.5, 4.1)	
25 – NH	7.81, d (7.6)	
β-OH-Asp		
26 – CO		168.7
27 – CH_α	4.70, t (6.0)	55.3
28 – CH_β	4.08, d (5.6)	71.3
29 – COOH		173.0
30 – NH	8.29, m	
Leu		
31 – CO		172.1
32 – CH_α	4.39, ovl*	50.8
33 – CH_β	1.43, ovl*	40.7
34 – CH_γ	1.40, ovl	24.0

35 – $CH_{\delta,1}$	0.80, d (5.9)	23.3
36 – $CH_{\delta,2}$	0.77, d (3.6)	21.2
37 – NH	8.18, d (8.2)	
Tyr		
38 – CO		171.1
39 – CH_{α}	4.47, m	54.3
40 – $CH_{\beta,1}$	2.84, dd (13.6, 5.2)	37.3
$CH_{\beta,2}$	2.61 dd (13.6, 3.8)	
41		127.6
42, 46	7.02, d (8.5)	130.2
43, 45	6.59, d (3.8)	114.7
44		155.8
44 – OH	9.12, bs	
47 – NH	8.32, d (8.2)	
Tyr		
48 – CO		171.3
49 – CH_{α}	4.38, ovl	54.5
50 – $CH_{\beta,1}$	2.55, dd (13.9, 4.0)	36.9
$CH_{\beta,2}$	2.37, dd (13.6, 9.9)	
51		128.1
52, 56	6.89, d (8.6)	130.0
53, 55	6.60, d (3.8)	114.8
54		155.7
54 – OH	9.12, bs	
57 – NH	7.97, bs	
Acetyl		
58 – CO		169.3
59	1.72, s	22.4

*signal overlapping

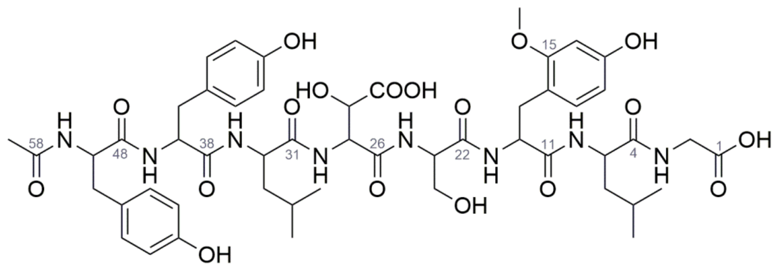

bosamycin D

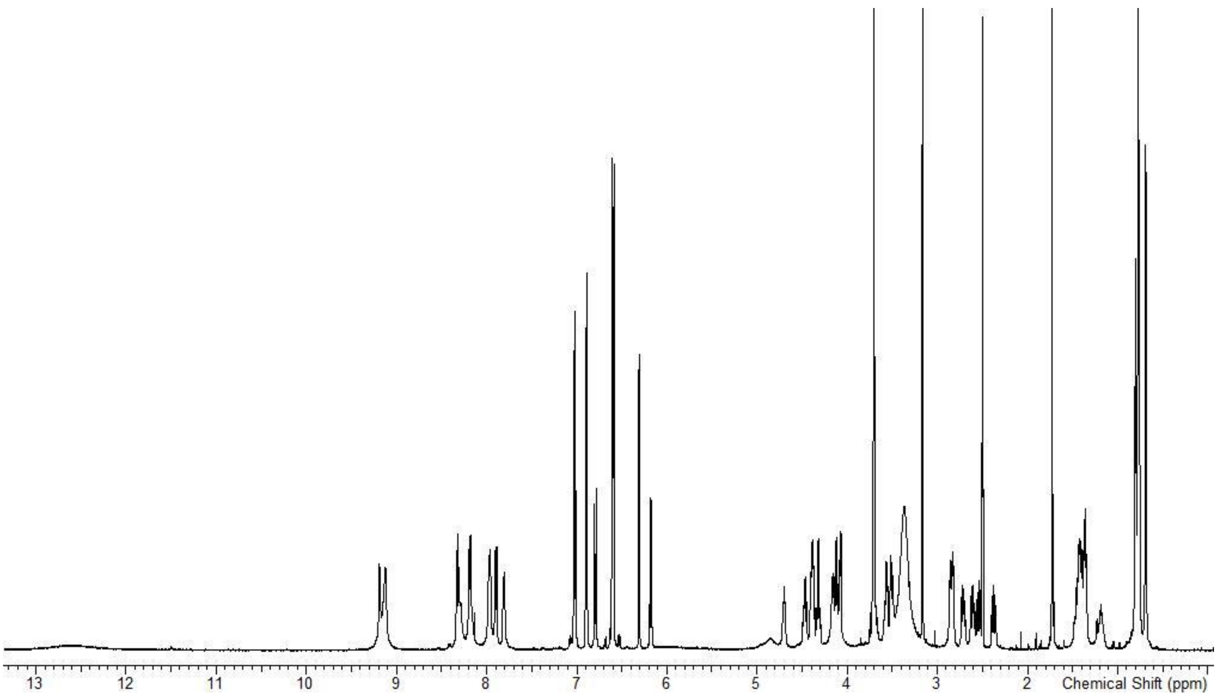

Figure S28: ^{1}H NMR spectrum (500 MHz, DMSO-*d6*) of bosamycin D.

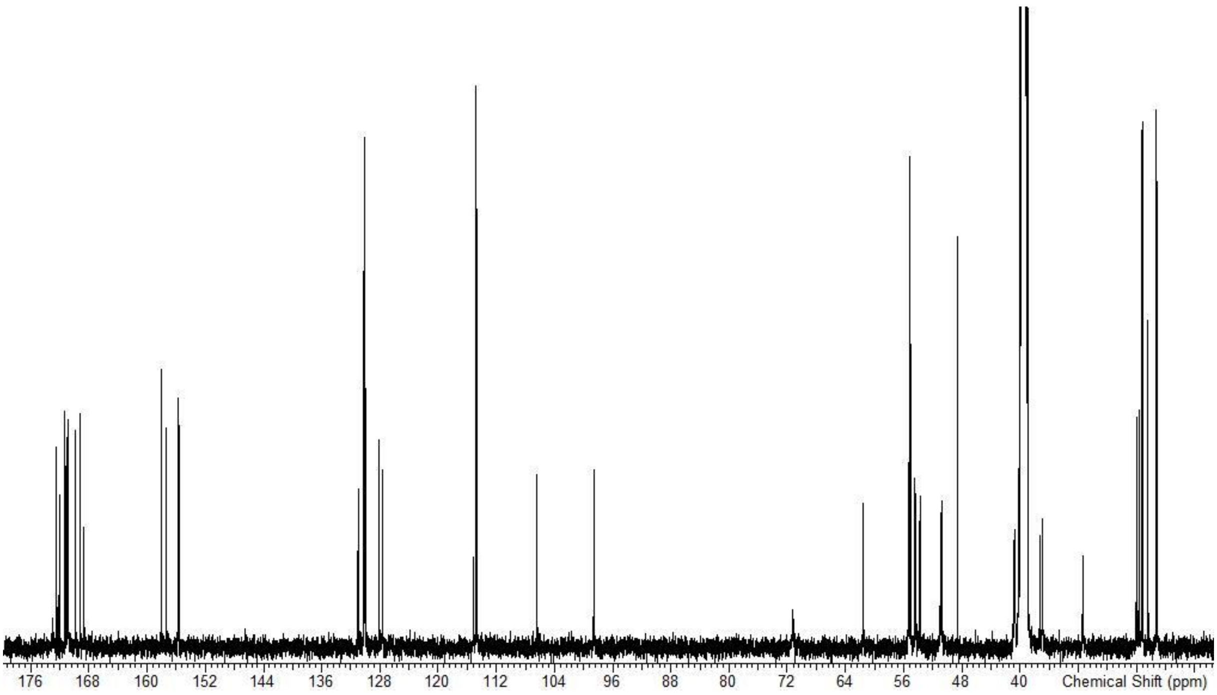

Figure S29: ^{13}C NMR spectrum (500 MHz, DMSO-*d6*) of bosamycin D.

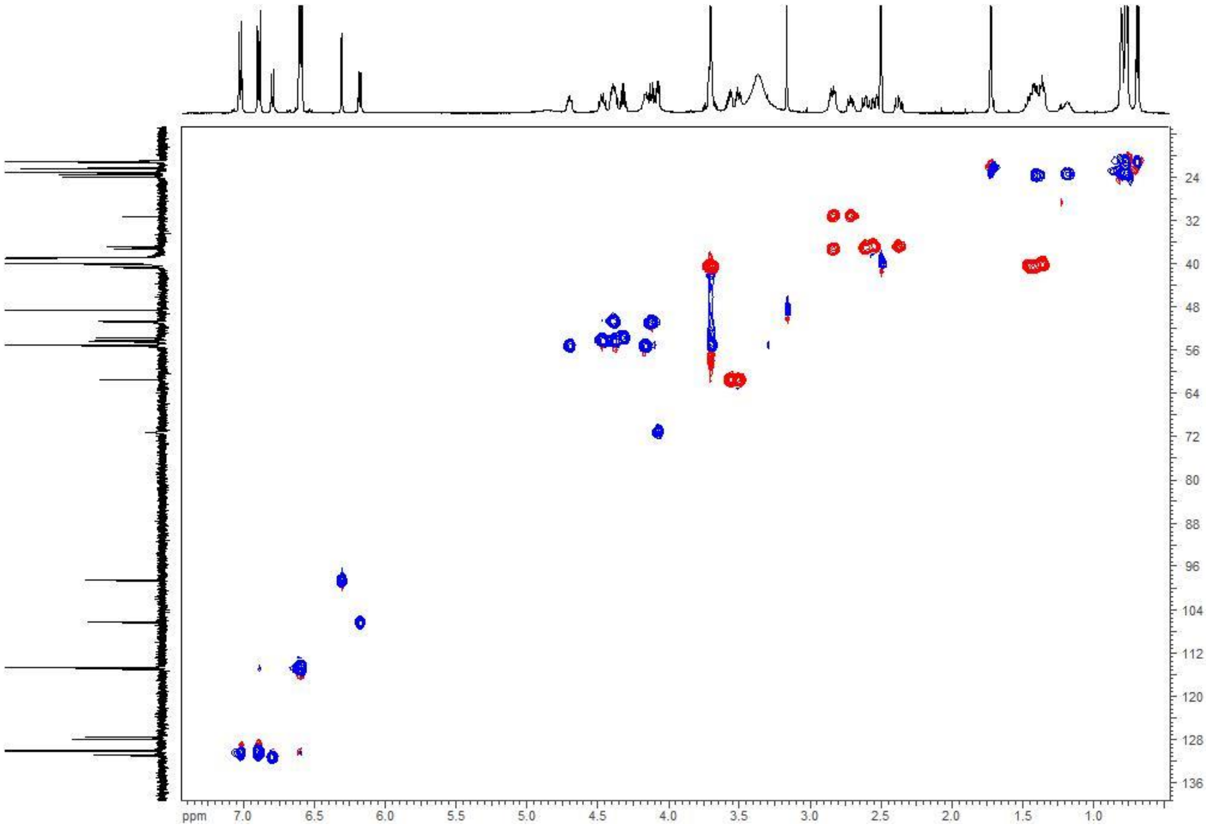

Figure S30: Edited HSQC spectrum (500 MHz, DMSO-*d6*) of bosamycin D.

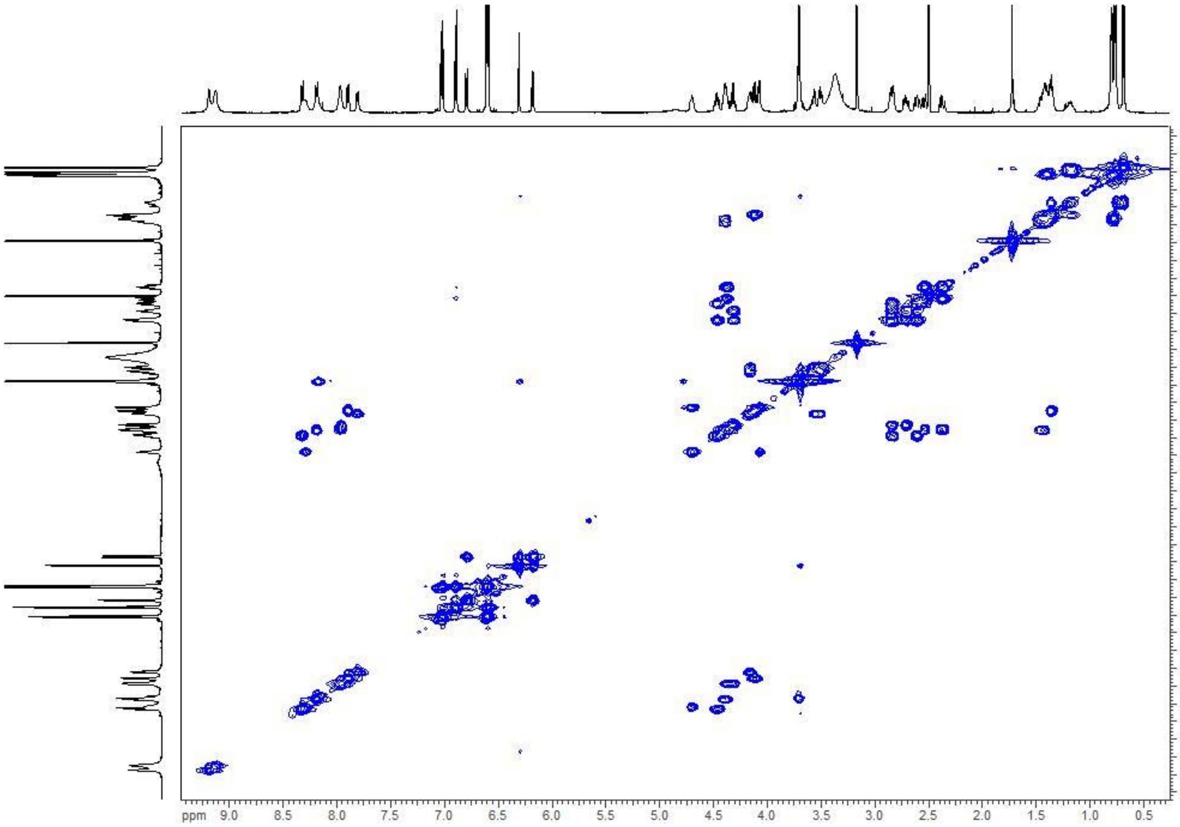

Figure S31: COSY spectrum (500 MHz, DMSO-*d6*) of bosamycin D.

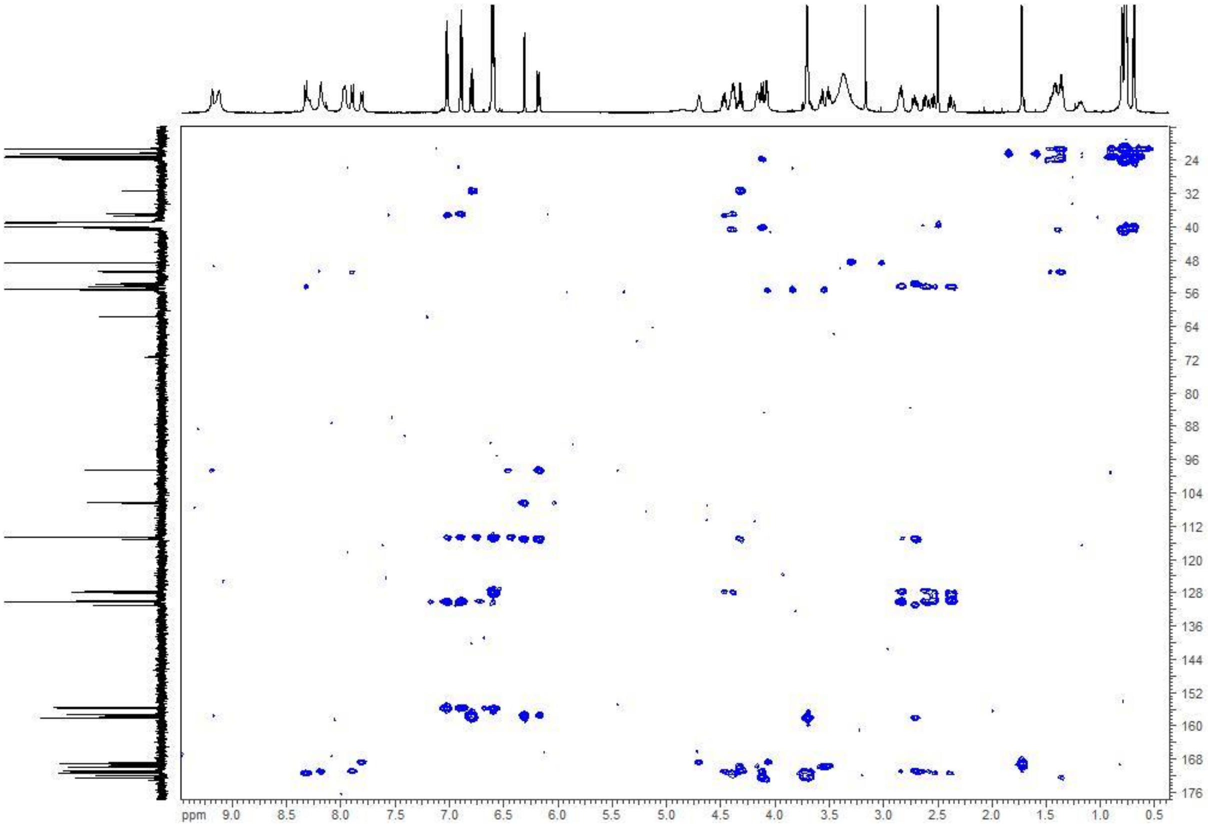

Figure S32: HMBC spectrum (500 MHz, DMSO-*d6*) of bosamycin D.

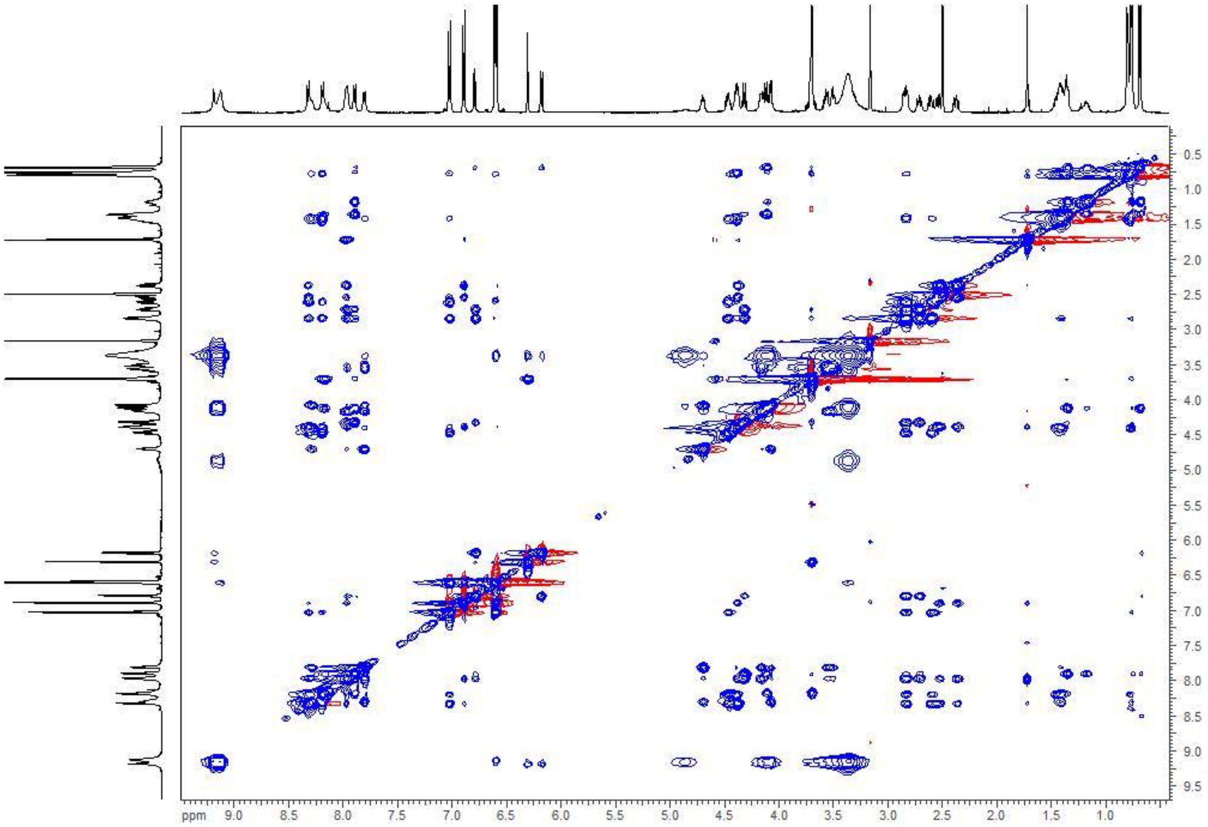

Figure S33: NOESY spectrum (500 MHz, DMSO-*d6*) of bosamycin D.

4. Stereochemical Assignment by Marfey's Method

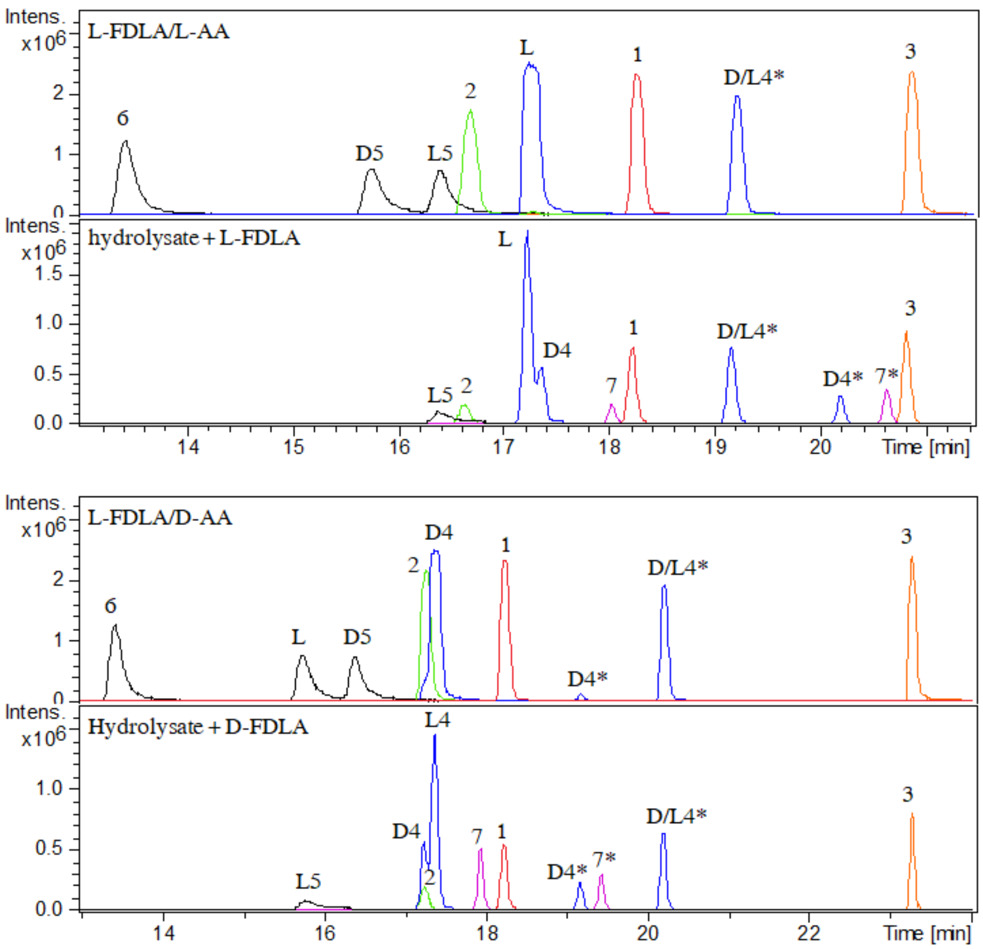

Figure S34: Marfey's method: MS spectra of bosamycin C derivatized with D-/L-FDLA (bottom) and the reference amino acids (AA) derivatized with D-/L-FDLA (top). The annotation is as follows: Gly (1), L-Ser (2), L-Leu (3), L-Tyr (L4, L4*), D-Tyr (D4, D4*), L-*erythro*-2-OH-Asp (L5), D-*erythro*-2-OH-Asp (D5), DL-*threo*-2-OH-Asp (6) and *o*-MeO-D-Tyr (7, 7*). L4*, D4* and 7* represent AA+2xFDLA. The exact location of D- and L-tyrosine was achieved later on by comparison of the biosynthetic gene cluster domains. 5-MeO Tyr was identified as D-conformer due to a shorter retention time when derivatized with D-FDLA

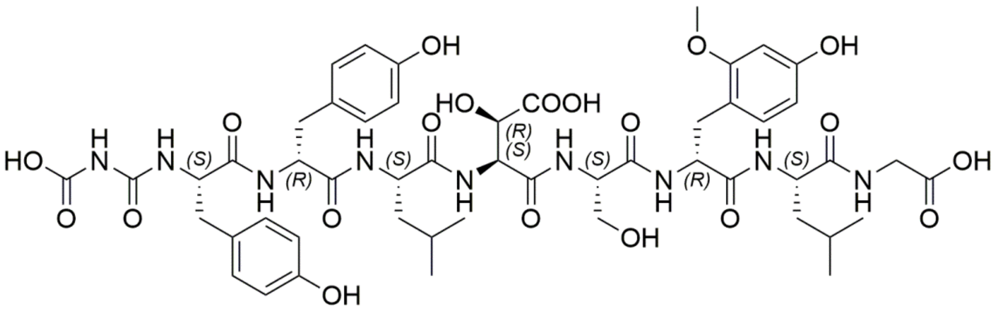

Figure S35: Structure of bosamycin C with stereochemistry determined by Marfey's method. The stereo chemical assignment of bosamycin C was in agreement with the data reported in the literature [3].

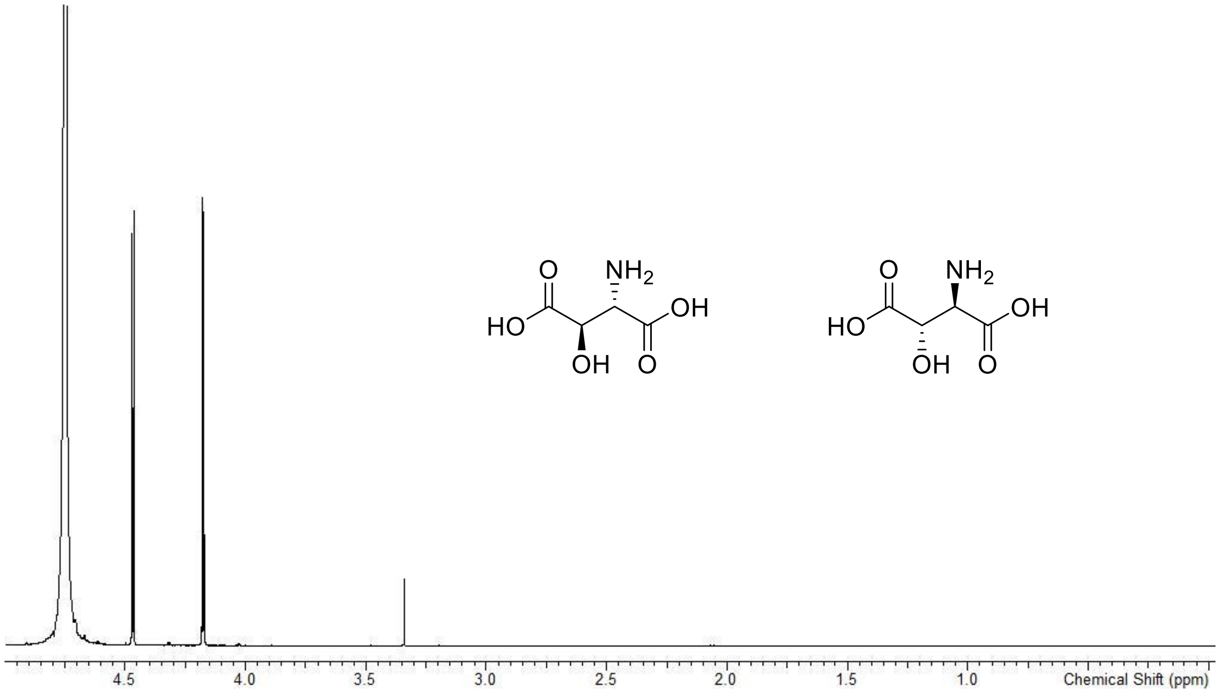

Figure S36: ^{1}H NMR spectrum (500 MHz, D_2O) of DL-*erythro*-*β*-hydroxyaspartic acid.

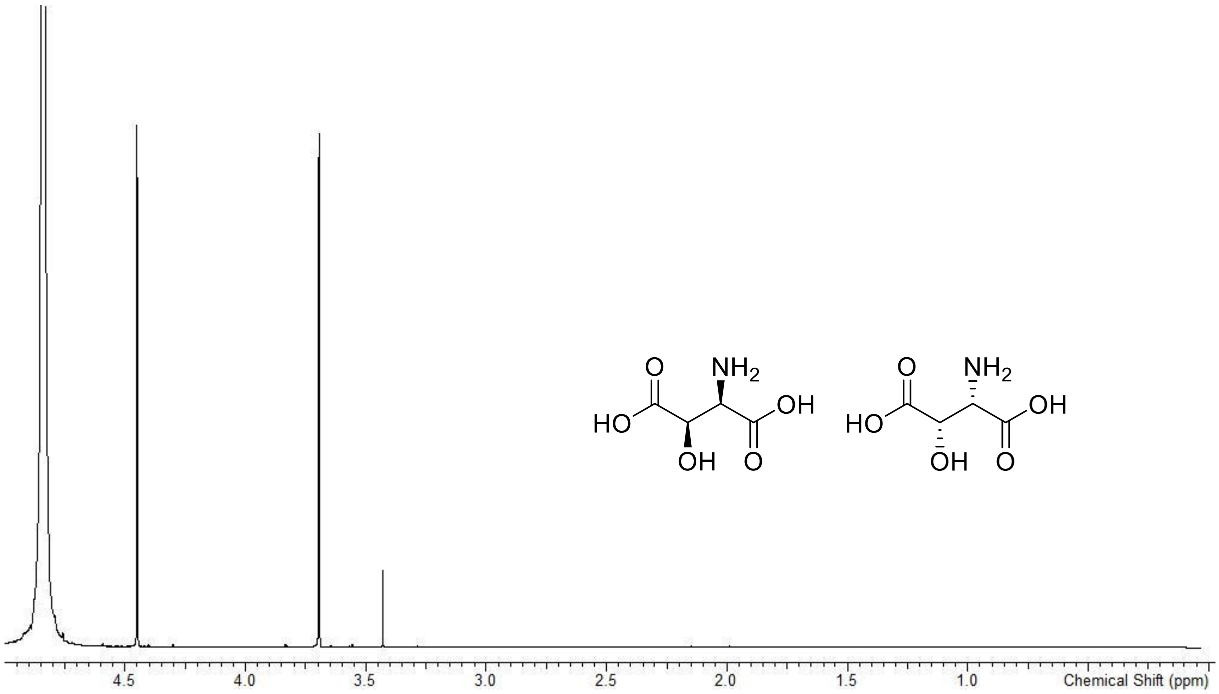

Figure S37: ^{1}H NMR spectrum (500 MHz, D_2O) of DL-*threo*-*β*-hydroxyaspartic acid.

5. MS/MS Fragmentation Data

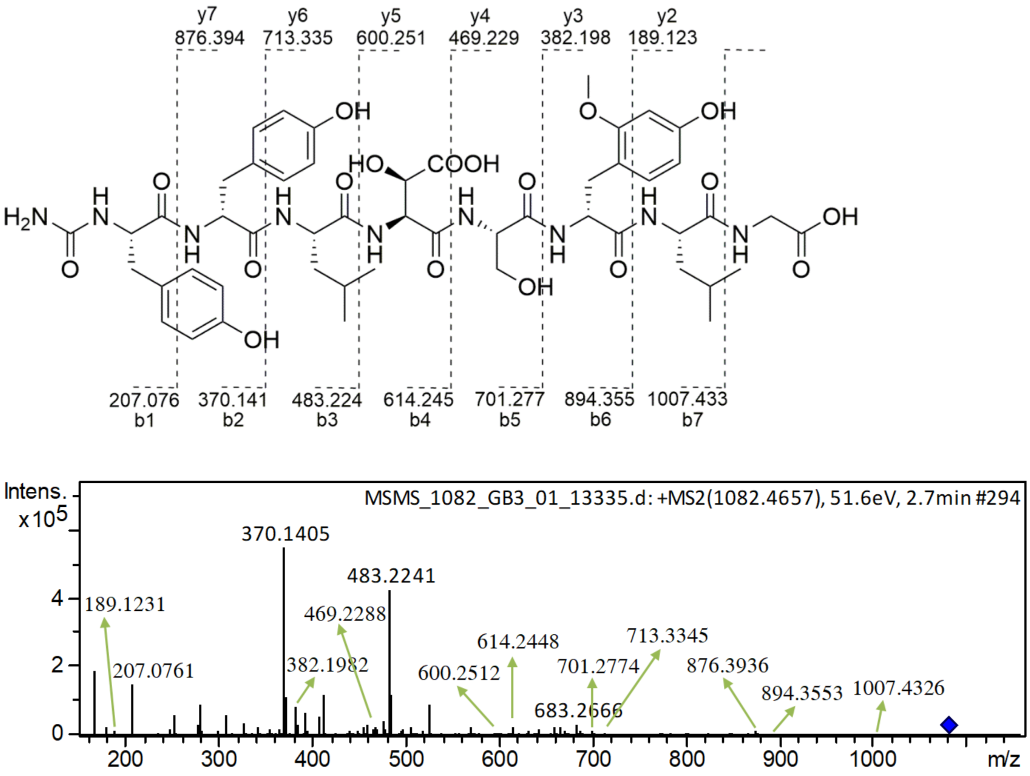

Figure S38: MS/MS analysis of bosamycin B.

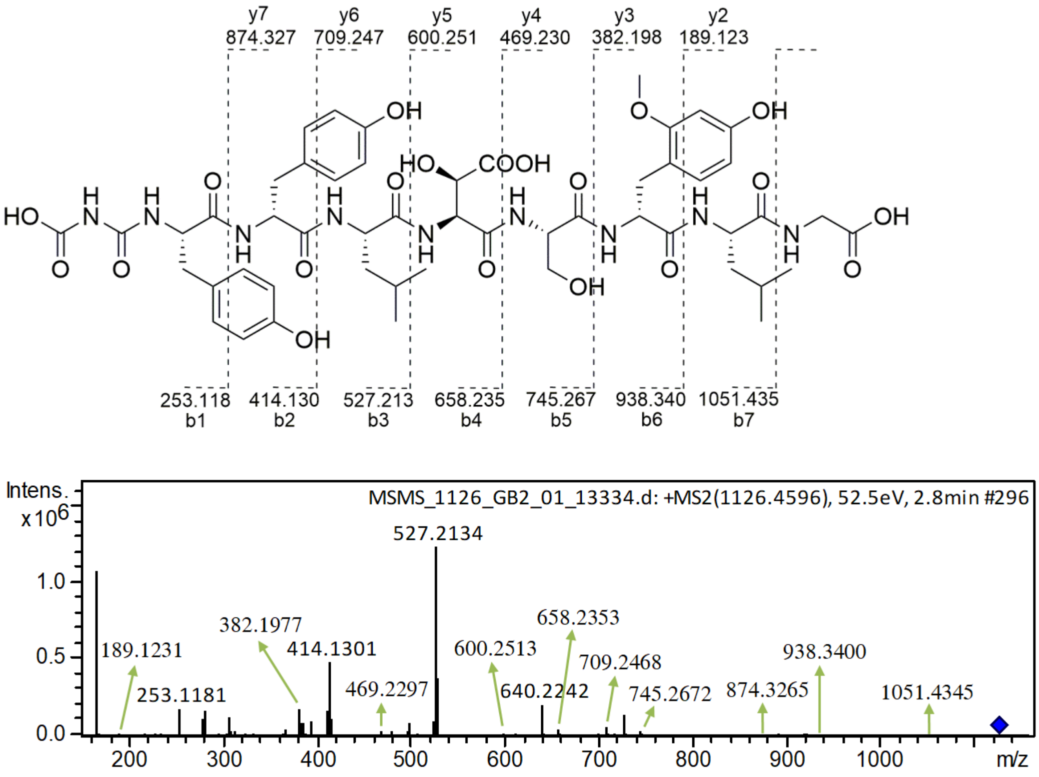

Figure S39: MS/MS analysis of bosamycin C.

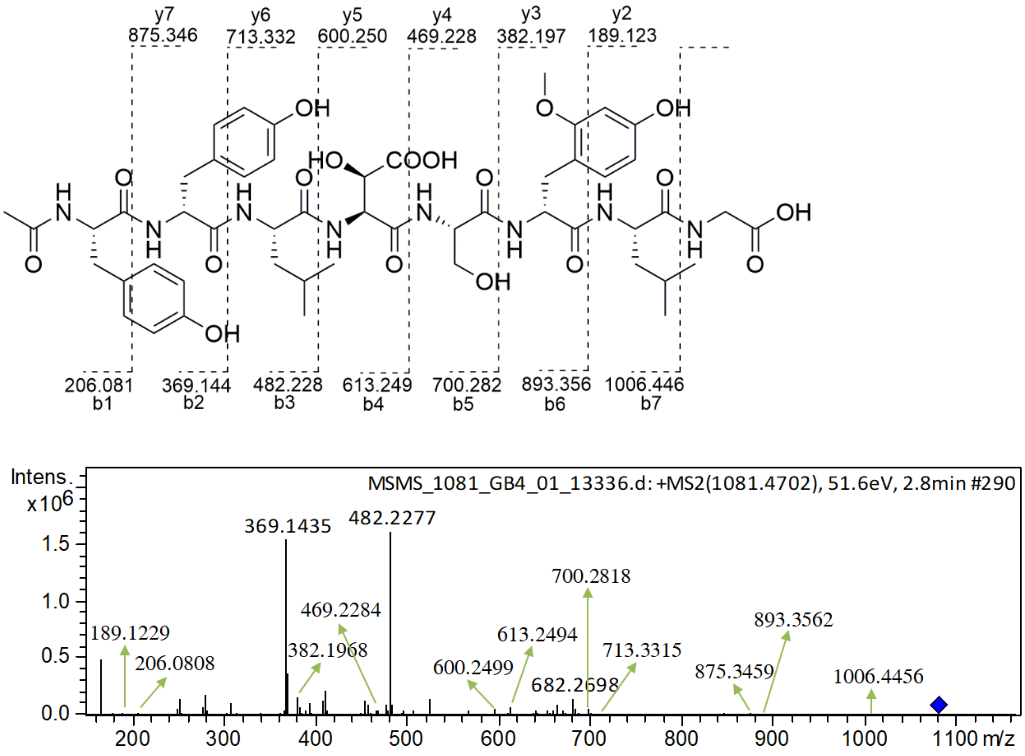

Figure S40: MS/MS analysis of bosamycin D.

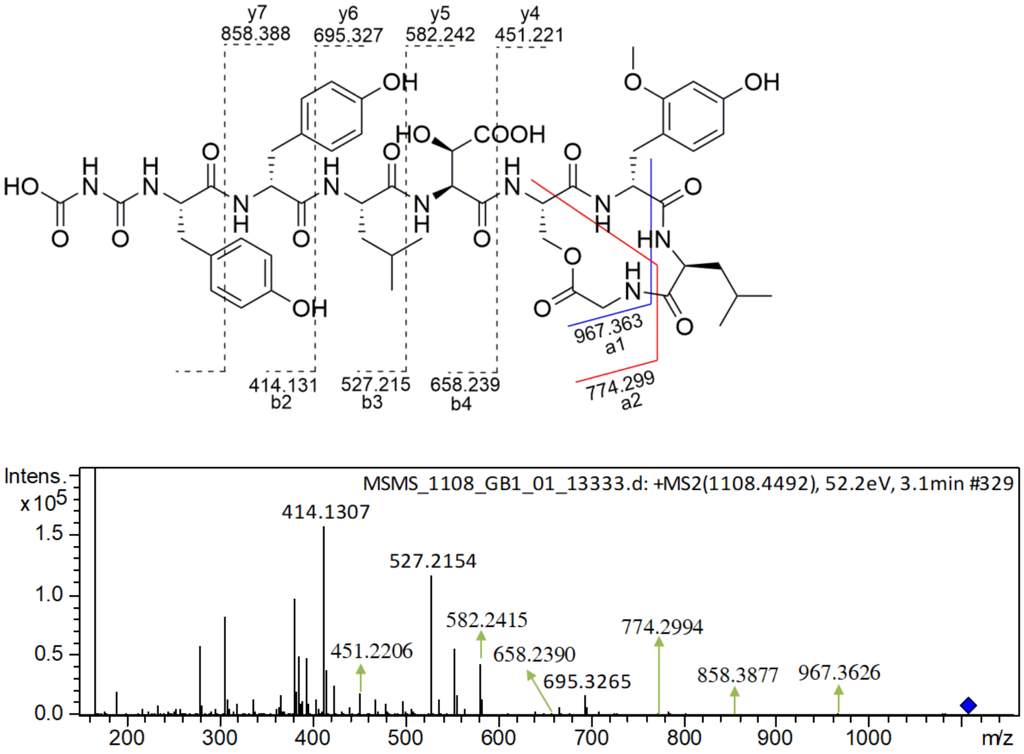

Figure S41: MS/MS analysis of depsibosamycin C.

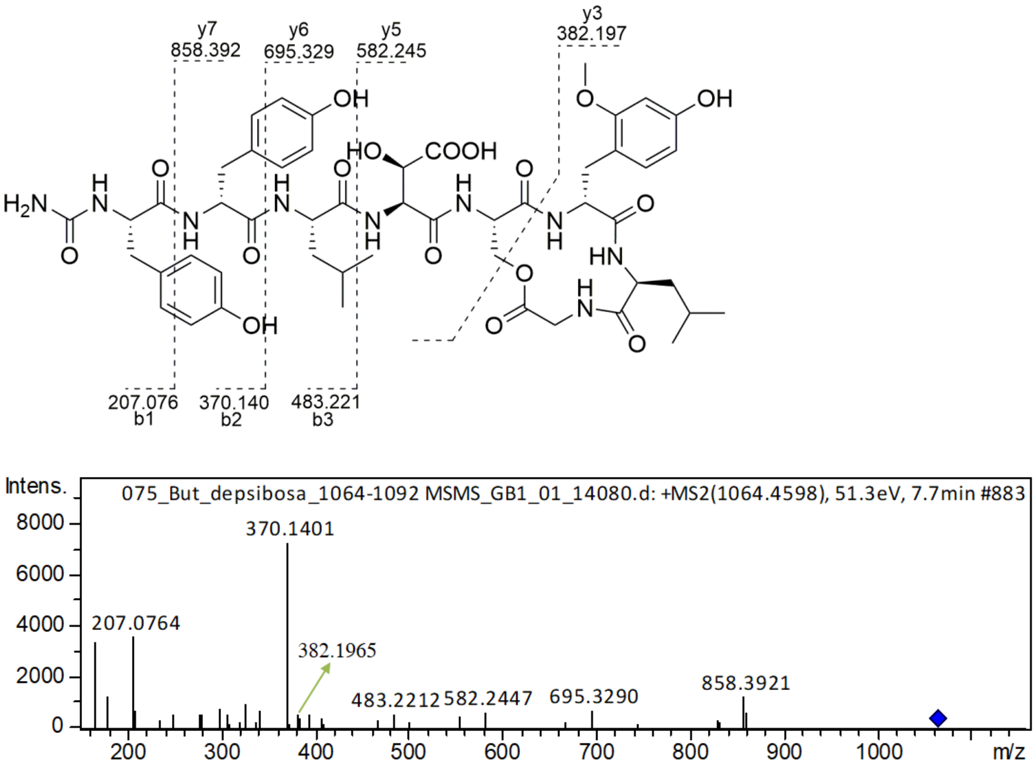

Figure S42: MS/MS of depsibosamycin B.

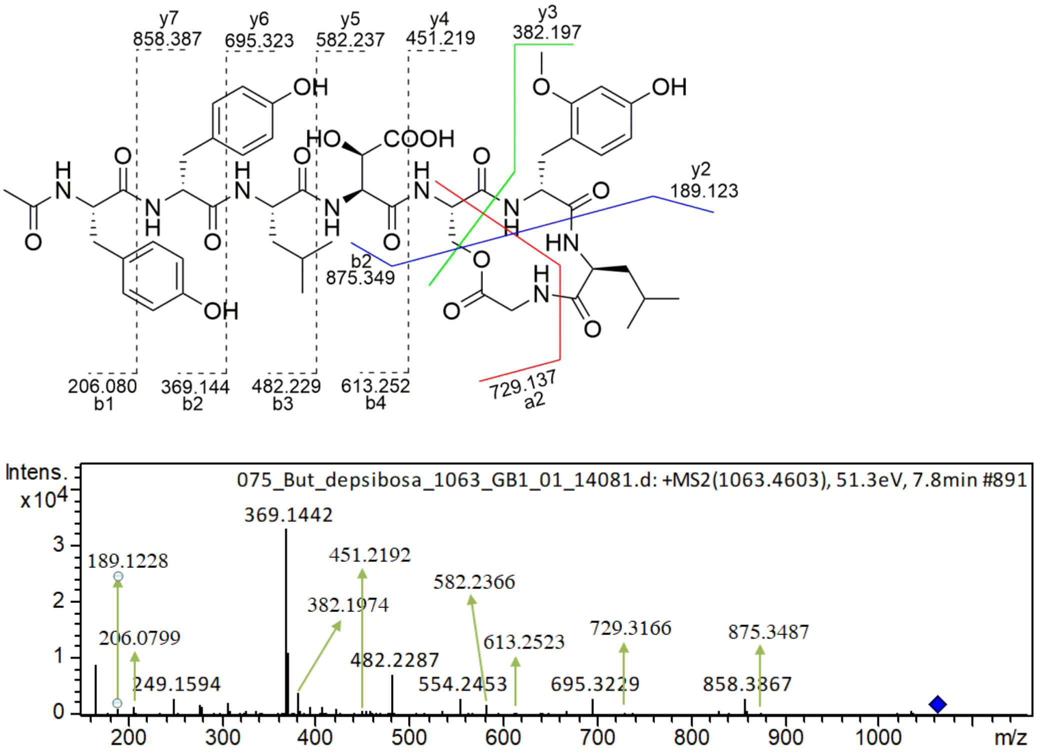

Figure S43: MS/MS analysis of depsibosamycin D.

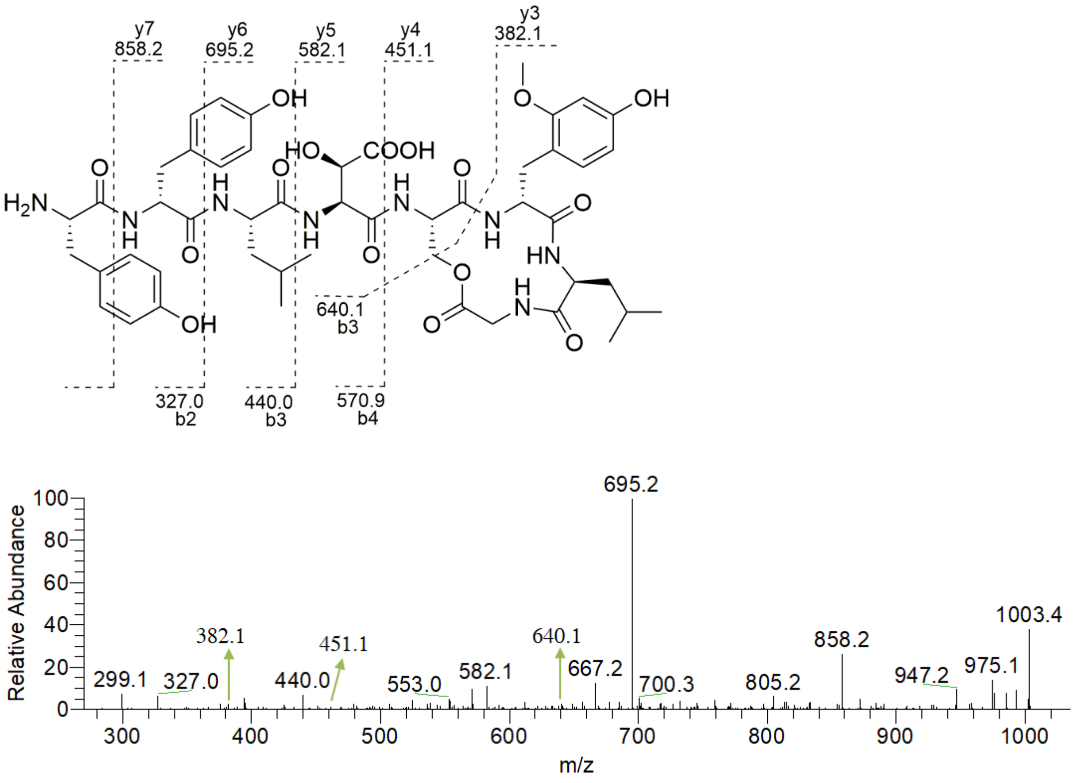

Figure S44: MS/MS of depsibosamycin N, produced by *S. lividans* I7.

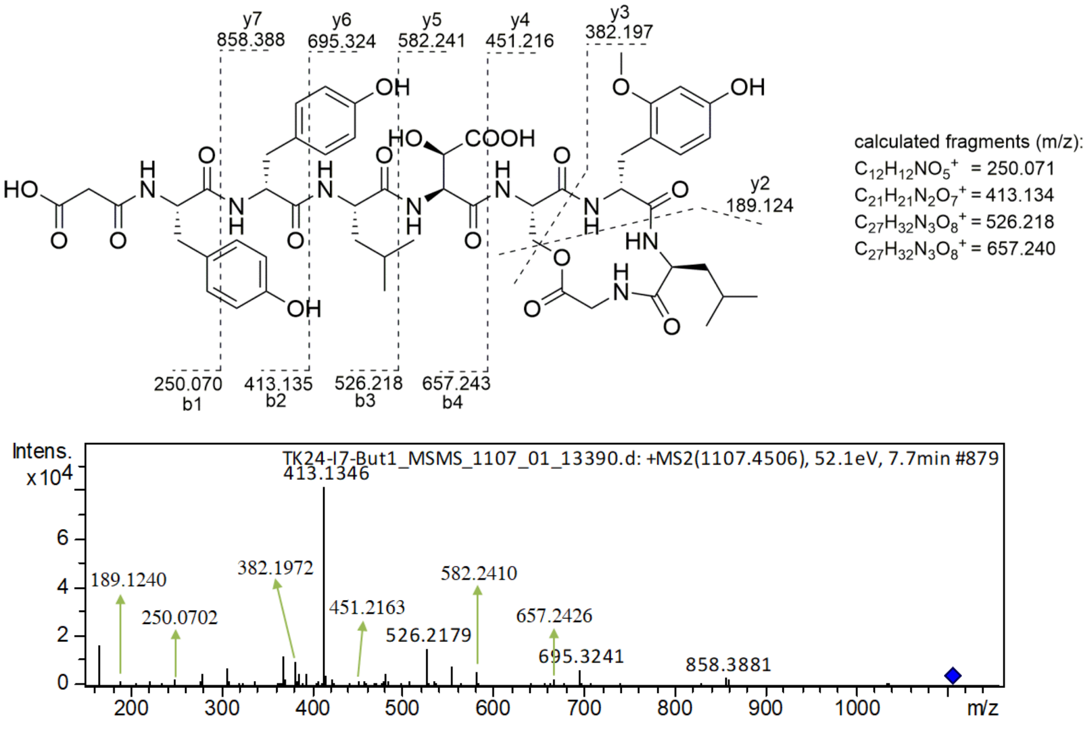

Figure S45: MS/MS analysis and the calculated masses of the expected fragments of depsibosamycin O produced by *S. lividans* I7.

Score	Expect	Method	Identities	Positives	Gaps
484 bits(1247)	5e-154	Compositional matrix adjust.	235/239(98%)	236/239(98%)	0/239(0%)

```
Query  1     PIFCVHPAGGHSWGYYHLKNHLPPGHPLYGLQARTLLQGAEPPATLAAMAADYVEQLRSV  60
             PIFCVHPAGGHSWGYYHLKNHLPPGHPLYGLQARTLLQGAEPPATLAAMAADYVEQLRSV
Sbjct  3676  PIFCVHPAGGHSWGYYHLKNHLPPGHPLYGLQARTLLQGAEPPATLAAMAADYVEQLRSV  3735

Query  61    QPHGPYHIVGWSFGGLVAFEMATRLQAQGEEVALLALLDAFPSAATEEGDEAVPADEDSM  120
             QPHGPYHIVGWSFGGLVAFEMATRLQAQGE VALLALLDAFPSAA EEGDEAVPADEDSM
Sbjct  3736  QPHGPYHIVGWSFGGLVAFEMATRLQAQGEVVALLALLDAFPSAAAEEGDEAVPADEDSM  3795

Query  121   LRMIAANAGYDPKDITTVGADEPLTPSALSEFFQQVGAVMANLTADDLRTFAHTLRHNAH  180
             LRMIAANAGYDPKDITTVGADEPLTPSALSEFFQQVGAVMANLTADDLRTFAHTLRHNAH
Sbjct  3796  LRMIAANAGYDPKDITTVGADEPLTPSALSEFFQQVGAVMANLTADDLRTFAHTLRHNAH  3855

Query  181   IGAAFTPGVFRGDVLLLTAALGRDGAETAPEKGRHAWTAHVTGRIDEHRVPCRHDDMLM  239
             IGAAFTPGVFRGDVLLLTAALGRDGAETAPEKGR AWTAHVTGRIDEHR+PCRHDDMLM
Sbjct  3856  IGAAFTPGVFRGDVLLLTAALGRDGAETAPEKGRRAWTAHVTGRIDEHRLPCRHDDMLM  3914
```

Figure S46: TE domain alignment of the depsibosamycin cluster (Query) and the bosamycin cluster (Sbjct).

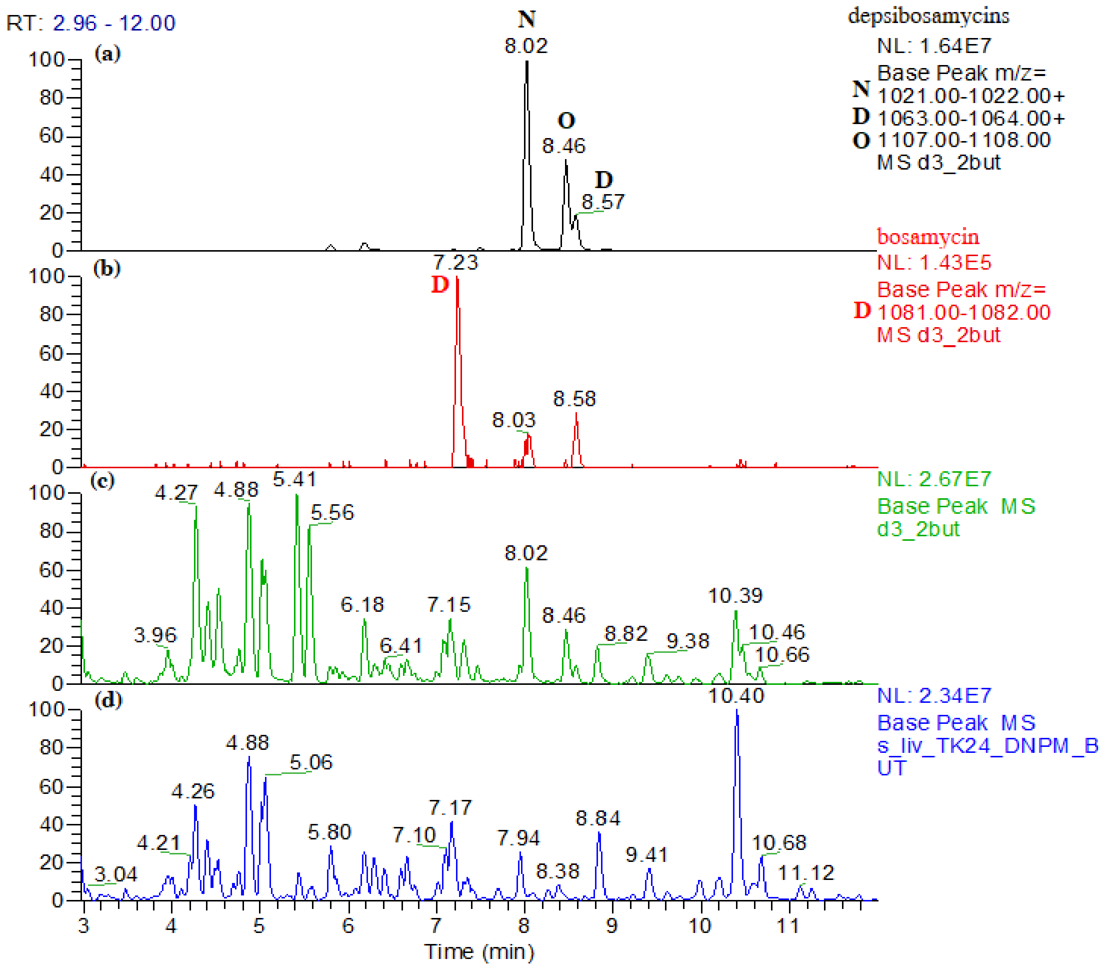

Figure S47: LC-MS chromatograms of *S. lividans* I7 with the dbm gene cluster showing (a) the extracted masses of depsibosamycin D, N and O (b) the extracted mass of bosamycin D, (c) the base peak chromatograms (bpc) of *S. lividans* I7 and (d) the bpc of the empty host *S. lividans* TK24 .

References

1. Rückert, C.; Albersmeier, A.; Busche, T.; Jaenicke, S.; Winkler, A.; Friðjónsson, Ó.H.; Hreggviðsson, G.Ó.; Lambert, C.; Badcock, D.; Bernaerts, K.; et al. Complete genome sequence of Streptomyces lividans TK24. *J. Biotechnol.* **2015**, *199*, 21-22, 10.1016/j.jbiotec.2015.02.004.
2. Flett, F.; Mersinias, V.; Smith, C.P. High efficiency intergeneric conjugal transfer of plasmid DNA from Escherichia coli to methyl DNA-restricting streptomycetes. *FEMS Microbiol. Lett.* **1997**, *155* (2), 223-229, 10.1111/j.1574-6968.1997.tb13882.x.
3. Xu, Z.F.; Bo, S.T.; Wang, M.J.; Shi, J.; Jiao, R.H.; Sun, Y.; Xu, Q.; Tan, R.X.; Ge, H.M. Discovery and biosynthesis of bosamycins from Streptomyces sp. 120454. *Chemical Science* **2020**, *11* (34), 9237-9245, 10.1039/D0NP90037K.
